# Monotone Circuit Lower Bounds from Robust Sunflowers

**DOI:** 10.1007/s00453-022-01000-3

**Published:** 2022-07-14

**Authors:** Bruno Pasqualotto Cavalar, Mrinal Kumar, Benjamin Rossman

**Affiliations:** 1 University of Warwick, Coventry, United Kingdom; 2 IIT Bombay, Mumbai, India; 3 Duke University, Durham, United States

**Keywords:** Monotone circuit complexity, Robust sunflower lemma, Sunflowers, Circuit complexity, Computational complexity, Extremal combinatorics, Monotone arithmetic circuits, Arithmetic circuit complexity

## Abstract

Robust sunflowers are a generalization of combinatorial sunflowers that have applications in monotone circuit complexity Rossman (SIAM J. Comput. 43:256–279, 2014), DNF sparsification Gopalan et al. (Comput. Complex. 22:275–310 2013), randomness extractors Li et al. (In: APPROX-RANDOM, LIPIcs 116:51:1–13, 2018), and recent advances on the Erdős-Rado sunflower conjecture Alweiss et al. (In: Proceedings of the 52nd Annual ACM SIGACT Symposium on Theory of Computing, STOC. Association for Computing Machinery, New York, NY, USA, 2020) Lovett et al. (From dnf compression to sunflower theorems via regularity, 2019) Rao (Discrete Anal. 8,2020). The recent breakthrough of Alweiss, Lovett, Wu and Zhang Alweiss et al. (In: Proceedings of the 52nd Annual ACM SIGACT Symposium on Theory of Computing, STOC. Association for Computing Machinery, New York, NY, USA, 2020) gives an improved bound on the maximum size of a *w*-set system that excludes a robust sunflower. In this paper, we use this result to obtain an $\exp (n^{1/2-o(1)})$ lower bound on the monotone circuit size of an explicit *n*-variate monotone function, improving the previous best known $\exp (n^{1/3-o(1)})$ due to Andreev (Algebra and Logic, 26:1–18, 1987) and Harnik and Raz (In: Proceedings of the Thirty-Second Annual ACM Symposium on Theory of Computing, ACM, New York, 2000). We also show an $\exp (\varOmega (n))$ lower bound on the monotone *arithmetic* circuit size of a related polynomial via a very simple proof. Finally, we introduce a notion of robust clique-sunflowers and use this to prove an $n^{\varOmega (k)}$ lower bound on the monotone circuit size of the CLIQUE function for all $k \leqslant n^{1/3-o(1)}$, strengthening the bound of Alon and Boppana (Combinatorica, 7:1–22, 1987).

## Introduction

A monotone Boolean circuit is a Boolean circuit with $\mathsf {AND}$ and $\mathsf {OR}$ gates but no negations ($\mathsf {NOT}$ gates). Although a restricted model of computation, monotone Boolean circuits seem a very natural model to work with when computing *monotone* Boolean functions, i.e., Boolean functions $f : \{0,1\}^n \rightarrow \{0,1\}$ such that for all pairs of inputs $(a_1, a_2, \ldots , a_n) , (b_1, b_2, \ldots , b_n)\in \{0,1\}^n$ where $a_i \leqslant b_i$ for every *i*, we have $f(a_1, a_2, \ldots , a_n) \leqslant f(b_1, b_2, \ldots , b_n)$. Many natural and well-studied Boolean functions such as $\mathsf {Clique}$ and $\mathsf {Majority}$ are monotone.

Monotone Boolean circuits have been very well studied in Computational Complexity over the years, and continue to be one of the few seemingly largest natural sub-classes of Boolean circuits for which we have exponential lower bounds. This line of work started with an influential paper of Razborov [23] from 1985 which proved an $n^{\varOmega (k)}$ lower bound on the size of monotone circuits computing the $\mathsf {Clique}_{k,n}$ function on *n*-vertex graphs for $k \leqslant \log n$; this bound is super-polynomial for $k = \log n$. Prior to Razborov’s result, super-linear lower bounds for monotone circuits were unknown, with the best bound being a lower bound of 4*n* due to Tiekenheinrich [29]. Further progress in this line of work included the results of Andreev [5] who proved an exponential lower bound for another explicit function. Alon and Boppana [2] extended Razborov’s result by proving an $n^{\varOmega (\sqrt{k})}$ lower bound for $\smash {\mathsf {Clique}_{k,n}}$ for all $k \leqslant n^{2/3 - o(1)}$. A second paper of Andreev [4] from the same time period proved an $2^{\varOmega (n^{1/3}/\log n)}$ lower bound for an explicit *n*-variate monotone function. Using a different technique, Harnik and Raz [12] proved a lower bound of $2^{\varOmega ((n/\log n)^{1/3})}$ for a family of explicit *n*-variate functions defined using a small probability space of random variables with bounded independence. However, modulo improvements to the polylog factor in this exponent, the state of art monotone circuit lower bounds have been stuck at $2^{\varOmega (n^{1/3 - o(1)})}$ since 1987.^1^ To this day, the question of proving truly exponential lower bounds for monotone circuits (of the form $2^{\varOmega (n)}$) for an explicit *n*-variate function) remains open! (Truly exponential lower bounds for monotone *formulas* were obtained only recently [20].)

In the present paper, we are able to improve the best known lower bound for monotone circuits by proving an $2^{\varOmega (n^{1/2}/\log n)}$ lower bound for an explicit *n*-variate monotone Boolean function (Sect. 2). The function is based on the same construction first considered by Harnik and Raz, but our argument employs the approximation method of Razborov with recent improvements on robust sunflower bounds [3, 21]. By applying the same technique with a variant of robust sunflowers that we call clique-sunflowers, we are able to prove an $n^{\varOmega (k)}$ lower bound for the $\mathsf {Clique}_{k,n}$ function when $k \leqslant n^{1/3-o(1)}$, thus improving the result of Alon and Boppana when *k* is in this range (Sect. 3). Finally, we are able to prove truly exponential lower bounds in the monotone arithmetic setting to a fairly general family of polynomials, which shares some similarities to the functions considered by Andreev and Harnik and Raz (Sect. 4).

### Monotone Circuit Lower Bounds and Sunflowers

The original lower bound for $\mathsf {Clique}_{k,n}$ due to Razborov employed a technique which came to be known as the *approximation method*. Given a monotone circuit *C* of “small size”, it consists into constructing gate-by-gate, in a bottom-up fashion, another circuit ${\widetilde{C}}$ that approximates *C* on most inputs of interest. One then exploits the structure of this *approximator circuit* to prove that it differs from $\mathsf {Clique}_{k,n}$ on most inputs of interest, thus implying that no “small” circuit can compute this function. This technique was leveraged to obtain lower bounds for a host of other monotone problems [2].

A crucial step in Razborov’s proof involved the sunflower lemma due to Erdős and Rado. A family ${\mathcal {F}}$ of subsets of [*n*] is called a *sunflower* if there exists a set *Y* such that $F_1 \cap F_2 = Y$ for every $F_1, F_2 \in {\mathcal {F}}$. The sets of ${\mathcal {F}}$ are called *petals* and the set $Y = \bigcap {\mathcal {F}}$ is called the *core*. We say that the family ${\mathcal {F}}$ is $\ell$-uniform if every set in the family has size $\ell$.

#### Theorem 1

(Erdős and Rado [8]) Let ${\mathcal {F}}$ be a $\ell$-uniform family of subsets of [*n*]. If $\left|{\mathcal {F}}\right| > \ell !(r-1)^\ell$, then ${\mathcal {F}}$ contains a sunflower of *r* petals.

Informally, the sunflower lemma allows one to prove that a monotone function can be approximated by one with fewer minterms by means of the “plucking” procedure: if the function has too many (more than $\ell !(r-1)^\ell$) minterms of size $\ell$, then it contains a sunflower with *r* petals; remove all the petals, replacing them with the core. One can then prove that this procedure does not introduce many errors.

The notion of *robust sunflowers* was introduced by the third author in [24], to achieve better bounds via the approximation method on the monotone circuit size of $\mathsf {Clique}_{k,n}$ when the negative instances are Erdős-Rényi random graphs ${\varvec{G}}_{n,p}$ below the *k*-clique threshold.^2^ A family ${\mathcal {F}} \subseteq 2^{[n]}$ is called a $(p, \varepsilon )$-*robust sunflower* if

$$\begin{aligned} {{\,\mathrm{\mathbb {P}}\,}}_{{\varvec{W}}\subseteq _p [n]} \left[ \exists F \in {\mathcal {F}}: F \subseteq {\varvec{W}}\cup Y \right] > 1-\varepsilon , \end{aligned}$$

where $Y := \bigcap {\mathcal {F}}$ and ${\varvec{W}}$ is a *p*-random subset of [*n*] (i.e., every element of [*n*] is contained in ${\varvec{W}}$ independently with probability *p*).

As remarked in [24], every $\ell$-uniform sunflower of *r* petals is a $(p, e^{-rp^{\ell }})$-robust sunflower. Moreover, as observed in [18], every (1/*r*, 1/*r*)-robust sunflower contains a sunflower of *r* petals. A corresponding bound for the appearance of robust sunflowers in large families was also proved in [24].

#### Theorem 2

([24]) Let ${\mathcal {F}}$ be a $\ell$-uniform family such that $\left|{\mathcal {F}}\right| \geqslant \ell !(2\log (1/\varepsilon )/p)^\ell$. Then ${\mathcal {F}}$ contains a $(p,\varepsilon )$-robust sunflower.

For many choice of parameters *p* and $\varepsilon$, this bound is better than the one by Erdős and Rado, thus leading to better approximation bounds. In a recent breakthrough, this result was significantly improved by Alweiss, Lovett, Wu and Zhang [3]. Soon afterwards, alternative proofs with slightly improved bounds were given by Rao^3^ [21] and Tao [28]. A more detailed discussion can be found in a note by Bell, Suchakree and Warnke [6].

#### Theorem 3

([3, 6, 21, 28]) There exists a constant $B > 0$ such that the following holds for all $p, \varepsilon \in (0,1/2]$. Let ${\mathcal {F}}$ be an $\ell$-uniform family such that $\left|{\mathcal {F}}\right| \geqslant (B \log (\ell /\varepsilon )/p)^\ell$. Then ${\mathcal {F}}$ contains a $(p,\varepsilon )$-robust sunflower.

Theorem 3 can be verified by combining the basic structure of Rossman’s original argument [24] with the main technical estimate of Rao [21]. Since the proof does not appear explicitly in any of those papers, for completeness we give a proof on Appendix A.

### Preliminaries

We denote by $\left\{ 0,1\right\} ^n_{= m} \subseteq \left\{ 0,1\right\} ^n$ the set of all *n*-bit binary vectors with Hamming weight exactly *m*. We extend the logical operators $\vee$ and $\wedge$ to binary strings $x, y \in \left\{ 0,1\right\} ^n$, as follows:

- $$(x \wedge y)_i = x_i \wedge y_i$$, for every $$i \in [n]$$;
- $$(x \vee y)_i = x_i \vee y_i$$, for every $$i \in [n]$$.

We will say that a distribution ${\varvec{X}}$ with support in $\left\{ 0,1\right\} ^n$ is *p*-*biased* or *p*-*random* if the random variables ${\varvec{X}}_1,\dots ,{\varvec{X}}_n$ are mutually independent and satisfy ${{\,\mathrm{\mathbb {P}}\,}}[{\varvec{X}}_i = 1] = p$ for all *i*. If a distribution ${\varvec{U}}$ has support in $2^{[n]}$, we will say that ${\varvec{U}}$ is *p*-*biased* or *p*-*random* if the random Boolean string ${\varvec{X}}$ such that ${\varvec{X}}_i = 1 \iff i \in {\varvec{U}}$ is *p*-biased. We sometimes write ${\varvec{U}}\subseteq _p [n]$ to denote that ${\varvec{U}}$ is a *p*-biased subset of [*n*].

We consistently write random objects using boldface symbols (such as ${\varvec{W}}$, ${\varvec{G}}_{n,p}$, etc). Everything that is not written in boldface is not random. When taking probabilities or expectation, the underlying distribution is always the one referred to by the boldface symbol. For instance, when $i \in [n]$ and ${\varvec{W}}$ is a *p*-biased subset of [*n*], the event $\left\{ i \in {\varvec{W}}\right\}$ denotes that the *non-random* element *i* is contained in the *random* set ${\varvec{W}}$.

For a Boolean function *f* and a probability distribution ${\varvec{\mu }}$ on the inputs on *f*, we write $f({\varvec{\mu }})$ to denote the random variable which evaluates *f* on a random instance of ${\varvec{\mu }}$.

In what follows, we will mostly ignore ceilings and floors for the sake of convenience, since these do not make any substantial difference in the final calculations.

## Harnik-Raz Function

The strongest lower bound known for monotone circuits computing an explicit *n*-variate monotone Boolean function is $\exp \big (\varOmega \big ((n/\log n)^{1/3}\big )\big )$, and it was obtained by Harnik and Raz [12]. In this section, we will prove a lower bound of $\exp (\varOmega (n^{1/2}/\log n))$ for the same Boolean function they considered. We apply the *method of approximations* [23] and the new *robust sunflower* bound [3, 21]. We do not expect that a lower bound better than $\exp (n^{1/2-o(1)})$ can be obtained by the approximation method with robust sunflowers. This limitation is discussed with more detail in Sect. 2.8.

We start by giving a high level outline of the proof. We define the Harnik-Raz function $f_\text {HR}: \left\{ 0,1\right\} ^n \rightarrow \left\{ 0,1\right\}$ and find two distributions ${\varvec{Y}}$ and ${\varvec{N}}$ with support in $\left\{ 0,1\right\} ^n$ satisfying the following properties:

- $f_\text {HR}$ outputs 1 on ${\varvec{Y}}$ with high probability (Lemma 1);
- $f_\text {HR}$ outputs 0 on ${\varvec{N}}$ with high probability (Lemma 2).

Because of these properties, the distribution ${\varvec{Y}}$ is called the *positive test distribution*, and ${\varvec{N}}$ is called the *negative test distribution*. We also define a set of monotone Boolean functions called *approximators*, and we show that:

- every approximator commits many mistakes on either ${\varvec{Y}}$ or ${\varvec{N}}$ with high probability (Lemma 9);
- every Boolean function computed by a “small” monotone circuit agrees with an approximator on both ${\varvec{Y}}$ and ${\varvec{N}}$ with high probability (Lemma 10).

Together these suffice for proving that “small” circuits cannot compute $f_\text {HR}$. The crucial part where the robust sunflower result comes into play is in the last two items.

### Notation for this Section

For $A \subseteq [n]$, let $x_A \in \left\{ 0,1\right\} ^n$ be the binary vector with support in *A*. For a set $A \subseteq [n]$, let ${\lceil }A{\rceil }$ be the indicator function satisfying

$$\begin{aligned} {\lceil }A{\rceil }(x) = 1 \iff x_A \leqslant x. \end{aligned}$$

For a monotone Boolean function $f : \left\{ 0,1\right\} ^n \rightarrow \left\{ 0,1\right\}$, let ${\mathcal M}(f)$ denote the set of minterms of *f*, and let ${\mathcal M}_\ell (f) := {\mathcal M}(f) \cap \left\{ 0,1\right\} _{=\ell }^n$. Elements of ${\mathcal M}_\ell (f)$ are called $\ell$-minterms of *f*.

This notation is valid only in Sect. 2 and will be slightly tweaked in Sect. 3 (Lower Bound for $\mathsf {Clique}_{k,n}$) for the sake of uniformity of exposition.

### The Function

We now describe the construction of the function $f_\text {HR}: \left\{ 0,1\right\} ^n \rightarrow \left\{ 0,1\right\}$ considered by Harnik and Raz [12]. First observe that, for every *n*-bit monotone Boolean function *f*, there exists a family ${\mathcal S}\subseteq 2^{[n]}$ such that

$$\begin{aligned} f(x_1,\dots ,x_n) = D_{\mathcal S}(x_1,\dots ,x_n) := \bigvee _{S \in {\mathcal S}} \bigwedge _{j \in S} x_{j}. \end{aligned}$$

Indeed, ${\mathcal S}$ can be chosen to be the family of the coordinate-sets of minterms of *f*. Now, in order to construct the Harnik-Raz function, we will suppose *n* is a prime number and let $\mathbb {F}_n = \left\{ 0,1,\dots ,n-1\right\}$ be the field of *n* elements. Moreover, we fix two positive integers *c* and *k* with $c< k < n$. For a polynomial $P \in \mathbb {F}_n[x]$, we let $S_P$ be the set of the valuations of *P* in each element of $\left\{ 1,2,\dots ,k\right\}$ (in other words, $S_P = \left\{ P(1),\dots ,P(k)\right\}$). Observe that it is not necessarily the case that $\left|S_P\right|=k$, since it may happen that $P(i)=P(j)$ for some *i*, *j* such that $i \ne j$. Finally, we consider the family ${\mathcal S}_\text {HR}$ defined as

$$\begin{aligned} {\mathcal S}_\text {HR}:= \left\{ S_P : P \in \mathbb {F}_n[x], P \text { has degree at most} c-1 \text { and} \left|S_P\right| \geqslant k/2 \right\} . \end{aligned}$$

We thus define $f_\text {HR}$ as $f_\text {HR}:= D_{{\mathcal S}_\text {HR}}$.

We now explain the choice of ${\mathcal S}_\text {HR}$. First, the choice for valuations of polynomials with degree at most $c-1$ is explained by a fact observed in [1]. If a polynomial ${\varvec{P}}\in \mathbb {F}_n[x]$ with degree $c-1$ is chosen uniformly at random, they observed that the random variables ${\varvec{P}}(1),\dots ,{\varvec{P}}(k)$ are *c*-wise independent, and are each uniform in [*n*]. This allows us to define a distribution on the inputs (the positive test distribution) that has high agreement with $f_\text {HR}$ and is easy to analyze. Observe further that, since $\left|{\mathcal S}_\text {HR}\right| \leqslant n^c$, the monotone complexity of $f_\text {HR}$ is at most $2^{O(c \log n)}$. Later we will choose *c* to be roughly $n^{1/2}$, and prove that the monotone complexity of $f_\text {HR}$ is $2^{\varOmega (c)}$.

Finally, the restriction $\left|S_P\right| \geqslant k/2$ is a truncation made to ensure that no minterm of $f_\text {HR}$ is very small. Otherwise, if $f_\text {HR}$ had small minterms, it might have been a function that almost always outputs 1. Such functions have very few maxterms and are therefore computed by a small CNF. Since we desire $f_\text {HR}$ to have high complexity, this is an undesirable property. The fact that $f_\text {HR}$ doesn’t have small minterms is important in the proof that $f_\text {HR}$ almost surely outputs 0 in the negative test distribution (Lemma 2).

#### Remark 1

(Parameters are now fixed) Formally, the function $f_\text {HR}$ depends on the choice of the parameters *c* and *k*. In other words, for every choice of positive integers *c*, *k* such that $c< k < n$, we obtain a different function $f_\text {HR}^{(c,k)}$. For the rest of Sect. 2, we will let *c* and *k* be fixed parameters, and we will refer to $f_\text {HR}$ unambiguously, always with respect to the fixed parameters *c* and *k*. We will make our choice of *c* and *k* explicit in Sect. 2.7, but before then we will make no assumptions about *c* and *k* other than $c< k < n$.

### Test Distributions

We now define the positive and negative test distributions.

#### Definition 1

*(Test distributions)* Let ${\varvec{Y}}\in \left\{ 0,1\right\} ^n$ be the random variable which chooses a polynomial ${\varvec{P}}\in \mathbb {F}_n[x]$ with degree at most $c-1$ uniformly at random, and maps it into the binary input $x_{S_{{\varvec{P}}}} \in \left\{ 0,1\right\} ^n$. Let also ${\varvec{N}}$ be the (1/2)-biased distribution on $\left\{ 0,1\right\} ^n$ (i.e., each bit is equal to 1 with probablity 1/2, independently of all the others). Equivalently, ${\varvec{N}}$ is the uniform distribution on $\left\{ 0,1\right\} ^n$.

Harnik and Raz proved that $f_\text {HR}$ outputs 1 on ${\varvec{Y}}$ with high probability. For completeness, we include their proof.

#### Lemma 1

(Claim 4.1 in [12]) We have ${{\,\mathrm{\mathbb {P}}\,}}[f_\text {HR}({\varvec{Y}})=1] \geqslant 1-(k-1)/n.$

#### Proof

Let ${\varvec{P}}$ be the polynomial randomly chosen by ${\varvec{Y}}$. Call a pair $\left\{ i,j\right\} \subseteq [k]$ with $i \ne j$ *coinciding* if ${\varvec{P}}(i) = {\varvec{P}}(j)$. Because the random variables ${\varvec{P}}(i)$ and ${\varvec{P}}(j)$ are uniformly distributed in [*n*] and independent for $i \ne j$, we have that ${{\,\mathrm{\mathbb {P}}\,}}[{\varvec{P}}(i) = {\varvec{P}}(j)] = 1/n$ for $i \ne j$. Therefore, the expected number $\mathsf {Num}({\varvec{P}})$ of coiciding pairs is $$\left( {\begin{array}{c}k\\ 2\end{array}}\right) /n$$. Observe now that $f_\text {HR}({\varvec{Y}}) = 0$ if and only if $\left|{\varvec{P}}(1), \dots , {\varvec{P}}(k)\right| < k/2$, which occurs only if there exists more than *k*/2 coinciding pairs. Therefore, by Markov’s inequality, we have

$$\begin{aligned} {{\,\mathrm{\mathbb {P}}\,}}\left[ f_\text {HR}({\varvec{Y}}) = 0 \right] \leqslant {{\,\mathrm{\mathbb {P}}\,}}\left[ \mathsf {Num}({\varvec{P}}) > k/2 \right] \leqslant \frac{\left( {\begin{array}{c}k\\ 2\end{array}}\right) /n}{k/2} = \frac{k-1}{n}. \end{aligned}$$

$$\square $$

We now claim that $f_\text {HR}$ also outputs 0 on ${\varvec{N}}$ with high probability.

#### Lemma 2

We have $$ {{\,\mathrm{\mathbb {P}}\,}}[f_\text {HR}({\varvec{N}})=0] \geqslant 1-2^{-(k/2-c\cdot \log _2 n)}. $$

#### Proof

Let $x_{{\varvec{A}}}$ be an input sampled from ${\varvec{N}}$. Observe that $f_\text {HR}(x_{{\varvec{A}}})=1$ only if there exists a minterm *x* of $f_\text {HR}$ such that $x \leqslant x_{{\varvec{A}}}$. Since all minterms of $f_\text {HR}$ have Hamming weight at least *k*/2 and $f_\text {HR}$ has at most $n^c$ minterms, we have

$$\begin{aligned} {{\,\mathrm{\mathbb {P}}\,}}[f_\text {HR}({\varvec{N}})=1] \leqslant n^c \cdot 2^{-k/2} = 2^{-(k/2-c\cdot \log _2 n)}. \end{aligned}$$

$$\square $$

We will also need the following property about the positive test distribution.

#### Lemma 3

For every $\ell \leqslant c$ and $A \subseteq [n]$ such that $\left|A\right|=\ell$, we have

$$\begin{aligned} {{\,\mathrm{\mathbb {P}}\,}}[x_A \leqslant {\varvec{Y}}] \leqslant \left( k/n \right) ^{\ell }. \end{aligned}$$

#### Proof

Recall that the distribution ${\varvec{Y}}$ takes a polynomial ${\varvec{P}}\in \mathbb {F}_n[x]$ with degree at most $c-1$ uniformly at random and returns the binary vector $x_{\left\{ {\varvec{P}}(1), {\varvec{P}}(2),\dots ,{\varvec{P}}(k)\right\} } \in \left\{ 0,1\right\} ^n$. Let $$A \in \left( {\begin{array}{c}[n]\\ \ell \end{array}}\right) $$ for $\ell \leqslant c$. Observe that $x_A \leqslant {{\varvec{Y}}}$ if and only if $A \subseteq \left\{ {\varvec{P}}(1), {\varvec{P}}(2),\dots ,{\varvec{P}}(k)\right\}$. Therefore, if $x_A \leqslant {\varvec{Y}}$, then there exists indices $\left\{ j_1,\dots ,j_\ell \right\}$ such that $\left\{ {\varvec{P}}(j_1), {\varvec{P}}(j_2),\dots ,{\varvec{P}}(j_{\ell })\right\} = A$. Since $\ell \leqslant c$, we get by the *c*-wise independence of ${\varvec{P}}(1),\dots ,{\varvec{P}}(k)$ that the random variables ${\varvec{P}}({j_1}), {\varvec{P}}({j_2}),\dots ,{\varvec{P}}({j_\ell })$ are independent. It follows that

$$\begin{aligned} {{\,\mathrm{\mathbb {P}}\,}}[ \left\{ {\varvec{P}}(j_1), {\varvec{P}}(j_2),\dots ,{\varvec{P}}(j_{\ell })\right\} = A ] = \frac{\ell !}{n^\ell }. \end{aligned}$$

Therefore, we have

$$\begin{aligned} {{\,\mathrm{\mathbb {P}}\,}}[ x_A \leqslant {\varvec{Y}}] = {{\,\mathrm{\mathbb {P}}\,}}[ A \subseteq \left\{ {\varvec{P}}(1), {\varvec{P}}(2),\dots ,{\varvec{P}}(k)\right\} ] \leqslant \left( {\begin{array}{c}k\\ \ell \end{array}}\right) \frac{\ell !}{n^\ell } \leqslant \left( \frac{k}{n} \right) ^{\ell }. \end{aligned}$$

$$\square $$

### A Closure Operator

In this section, we describe a closure operator in the lattice of monotone Boolean functions. We prove that the closure of a monotone Boolean function *f* is a good approximation for *f* on the negative test distribution (Lemma 4), and we give a bound on the size of the set of minterms of *closed* monotone functions. This bound makes use of the robust sunflower lemma (Theorem 3), and is crucial to bounding errors of approximation (Lemma 8). Finally, we observe that input functions are closed (Lemma 6). From now on, we let

1 $$\begin{aligned} \varepsilon := n^{-2c}. \end{aligned}$$

#### Definition 2

*(Closed function)* We say that a monotone function $f : \left\{ 0,1\right\} ^n \rightarrow \left\{ 0,1\right\}$ is *closed* if, for every $$A \in \left( {\begin{array}{c}[n]\\ \leqslant c\end{array}}\right) $$, we have

$$\begin{aligned} {{\,\mathrm{\mathbb {P}}\,}}[\ f({\varvec{N}}\vee x_A) = 1\ ] > 1 - \varepsilon \ \Longrightarrow \ f(x_A) = 1. \end{aligned}$$

This means that for, a closed function, we always have ${{\,\mathrm{\mathbb {P}}\,}}[f({\varvec{N}}\vee x_A) = 1] \notin (1-\varepsilon ,1)$ when $\left|A\right| \leqslant c$.

#### Remark 2

[On the parametrization of closedness] We remark that the definition of a *closed* function depends on two parameters: the parameter $\varepsilon$, defined in (1), and the parameter *c*, used in the construction of $f_\text {HR}$ (see Remark 1). Since both of these parameters are fixed throughout Sect. 2, it is safe to omit them without risk of confusion. Therefore, we will henceforth say that some function is *closed* without any further specification about the parameters. However, the reader must bear in mind that, whenever a function is said to be *closed*, the *fixed* parameters *c* and $\varepsilon$ are in view.

#### Definition 3

*(Closure operator)* Let *f* be a monotone Boolean function. We denote by $\mathrm {cl}(f)$ the unique minimal closed monotone Boolean function such that $f \leqslant \mathrm {cl}(f)$. In other words, the function $\mathrm {cl}(f)$ is the unique closed monotone function such that, whenever $f \leqslant g$ and *g* is monotone and closed, we have $f \leqslant \mathrm {cl}(f) \leqslant g$.

#### Remark 3

(On closure) Note that $\mathrm {cl}(f)$ is well-defined, since the constant Boolean function that outputs 1 is closed and, if *f*, *g* are both closed monotone Boolean functions, then so is $f \wedge g$. Furthermore, just as with the definition of closed functions (see Remark 2), the closure operator $\mathrm {cl}(\cdot )$ depends crucially on the parameters $\varepsilon$ and *c*, which are fixed throughout Sect. 2.

We now give a bound on the error of approximating *f* by $\mathrm {cl}(f)$ under the distribution ${\varvec{N}}$.

#### Lemma 4

(Approximation by closure) For every monotone $f: \left\{ 0,1\right\} ^n \rightarrow \left\{ 0,1\right\}$, we have

$$\begin{aligned} {{\,\mathrm{\mathbb {P}}\,}}\left[ f({\varvec{N}}) = 0 \text { and } \mathrm {cl}(f)({\varvec{N}}) = 1 \right] \leqslant n^{-c}. \end{aligned}$$

#### Proof

We first prove that there exists a positive integer *t* and sets $A_1, \dots , A_t$ and monotone functions $h_0, h_1, \dots , h_t : \left\{ 0,1\right\} ^n \rightarrow \left\{ 0,1\right\}$ such that

1. $$h_0 = f$$,
2. $$h_i = h_{i-1} \vee {\lceil }A_i{\rceil }$$,
3. $${{\,\mathrm{\mathbb {P}}\,}}[h_{i-1}({\varvec{N}}\vee x_{A_i}) = 1] \geqslant 1-\varepsilon $$,
4. $$h_t = \mathrm {cl}(f)$$.

Indeed, if $h_{i-1}$ is not closed, there exists $$A_i \in \left( {\begin{array}{c}[n]\\ \leqslant c\end{array}}\right) $$ such that ${{\,\mathrm{\mathbb {P}}\,}}[h_{i-1}({\varvec{N}}\vee x_{A_i}) = 1] \geqslant 1-\varepsilon$ but $h_{i-1}(x_{A_i})=0$. We let $h_i := h_{i-1} \vee {\lceil }A_i{\rceil }$. Clearly, we have that $h_t$ is closed, and that the value of *t* is at most the number of subsets of [*n*] of size at most *c*. Therefore, we get $$ t \leqslant \sum _{j=0}^{c} \left( {\begin{array}{c}n\\ j\end{array}}\right) . $$ Moreover, by induction we obtain that $h_i \leqslant \mathrm {cl}(f)$ for every $i \in [t]$. It follows that $h_t = \mathrm {cl}(f)$. Now, observe that

$$\begin{aligned} {{\,\mathrm{\mathbb {P}}\,}}\left[ f({\varvec{N}}) = 0 \text { and } \mathrm {cl}(f)({\varvec{N}}) = 1 \right]&\leqslant \sum _{i=1}^t {{\,\mathrm{\mathbb {P}}\,}}\left[ h_{i-1}({\varvec{N}}) = 0 \text { and } h_i({\varvec{N}}) = 1 \right] \\ {}&= \sum _{i=1}^t {{\,\mathrm{\mathbb {P}}\,}}\left[ h_{i-1}({\varvec{N}}) = 0 \text { and } x_{A_i} \leqslant {\varvec{N}}\right] \\ {}&\leqslant \sum _{i=1}^t {{\,\mathrm{\mathbb {P}}\,}}\left[ h_{i-1}({\varvec{N}}\vee x_{A_i}) = 0 \right] \\ {}&\leqslant \varepsilon \sum _{j=0}^{c} \left( {\begin{array}{c}n\\ j\end{array}}\right) \leqslant n^{-c}. \end{aligned}$$

$$\square $$

We now bound the size of the set of $\ell$-minterms of a closed function. This bound depends on the robust sunflower theorem (Theorem 3).

#### Lemma 5

(Closed functions have few minterms) Let $B > 0$ be as in Theorem 3. If a monotone function $f : \left\{ 0,1\right\} ^n \rightarrow \left\{ 0,1\right\}$ is closed, then, for all $\ell \in [c]$, we have

$$\begin{aligned} \left|{\mathcal M}_\ell (f)\right| \leqslant (6B c \log n)^\ell \end{aligned}$$

#### Proof

Fix $\ell \in [c]$. For convenience, let $p=1/2$ and recall from (3) that $\varepsilon =n^{-2c}$. We will begin by proving that $\left|{\mathcal M}_\ell (f)\right| \leqslant (B \log (\ell /\varepsilon )/p)^\ell .$

For a contradiction, suppose we have $\left|{\mathcal M}_\ell (f)\right| > (B \log (\ell /\varepsilon )/p)^\ell .$ Consider the family $${\mathcal {F}} := \left\{ A \in \left( {\begin{array}{c}[n]\\ \ell \end{array}}\right) : x_A \in {\mathcal M}_\ell (f)\right\} $$. Observe that $\left|{\mathcal {F}}\right| = \left|{\mathcal M}_\ell (f)\right|$. By Theorem 3, there exists a $(p,\varepsilon )$-robust sunflower ${\mathcal {F}}' \subseteq {\mathcal {F}}$. Let $Y := \bigcap {\mathcal {F}}'$ and let ${\varvec{W}}\subseteq _p [n]$. We have

$$\begin{aligned} {{\,\mathrm{\mathbb {P}}\,}}[f({\varvec{N}}\vee x_Y)=1]&\geqslant {{\,\mathrm{\mathbb {P}}\,}}[\exists x \in {\mathcal M}_\ell (f) : x \leqslant {\varvec{N}}\vee x_Y] \\ {}&= {{\,\mathrm{\mathbb {P}}\,}}[\exists F \in {\mathcal {F}} : F \subseteq {\varvec{W}}\cup Y ] \\ {}&\geqslant {{\,\mathrm{\mathbb {P}}\,}}[\exists F \in {\mathcal {F}}' : F \subseteq {\varvec{W}}\cup Y ] \\ {}&> 1-\varepsilon . \end{aligned}$$

Therefore, since *f* is closed, we get that $f(x_Y)=1$. However, since $Y = \bigcap {\mathcal {F}}'$, there exists $F \in {\mathcal {F}}'$ such that $Y \subsetneq F$. This is a contradiction, because $x_F$ is a minterm of *f*. We conclude that

$$\begin{aligned} \left|{\mathcal M}_\ell (f)\right| \leqslant (B \log (\ell /\varepsilon )/p)^\ell \leqslant (2B \log (cn^{2c}))^\ell \leqslant (6B c \log n)^\ell . \end{aligned}$$

$$\square $$

#### Lemma 6

(Input functions are closed) For all $i \in [n]$ and large enough *n*, the Boolean functions ${\lceil }\left\{ i\right\} {\rceil }$ are closed.

#### Proof

Fix $i \in [n]$. Let $A \subseteq [n]$ be such that $\left|A\right| \leqslant c$ and suppose that ${\lceil }\left\{ i\right\} {\rceil }(x_A) = 0$. Note that ${\lceil }\left\{ i\right\} {\rceil }(x_A) = 0$ is equivalent to $(x_A)_i = 0$. We have

$$\begin{aligned} {{\,\mathrm{\mathbb {P}}\,}}[{\lceil }\left\{ i\right\} {\rceil }({\varvec{N}}\vee x_A)=1] = {{\,\mathrm{\mathbb {P}}\,}}[({\varvec{N}}\vee x_A)_i = 1] = {{\,\mathrm{\mathbb {P}}\,}}[{\varvec{N}}_i = 1] = 1/2 \leqslant 1-n^{-2c} = 1-\varepsilon , \end{aligned}$$

since ${\varvec{N}}$ is (1/2)-biased (Definition 1) and $\varepsilon = n^{-2c}$ (as fixed in (3)). Therefore, ${\lceil }\left\{ i\right\} {\rceil }$ is closed. $\square$

### Trimmed Monotone Functions

In this section, we define a *trimming* operation for Boolean functions. We will bound the probability that a *trimmed* function gives the correct output on the distribution ${\varvec{Y}}$, and we will give a bound on the error of approximating a Boolean function *f* by the trimming of *f* on that same distribution.

#### Definition 4

*(Trimmed functions)* We say that a monotone function $f \in \left\{ 0,1\right\} ^n \rightarrow \left\{ 0,1\right\}$ is *trimmed* if all the minterms of *f* have size at most *c*/2. We define the trimming operation ${{\,\mathrm{trim}\,}}(f)$ as follows:

$$\begin{aligned} {{\,\mathrm{trim}\,}}(f) := \bigvee _{\ell = 0}^{{c/2}} \bigvee _{A \in {\mathcal M}_{\ell }(f)} {\lceil }A{\rceil }. \end{aligned}$$

That is, the ${{\,\mathrm{trim}\,}}$ operation takes out from *f* all the minterms of size larger than *c*/2, yielding a trimmed function.

#### Remark 4

(Parametrization of ${{\,\mathrm{trim}\,}}(\cdot )$ and other remarks) We remark that the definition of trimmed functions depends on the choice of the parameter *c*. As this parameter is fixed (see Remark 1), the operator ${{\,\mathrm{trim}\,}}(\cdot )$ is well-defined. Moreover, if all minterms of *f* have Hamming weight larger than *c*/2 (i.e., if ${\mathcal M}_{\ell }(f) = \emptyset$ for all $\ell \in \left\{ 0,1,\dots ,c/2\right\}$), then ${{\,\mathrm{trim}\,}}(f)$ is the constant function that outputs 0. Finally, if *f* is the constant function $\mathbbm {1}$, then ${{\,\mathrm{trim}\,}}(f) = \mathbbm {1}$, because $\mathbbm {1}$ contains a minterm of Hamming weight equal to 0.

We are now able to bound the probability that a trimmed Boolean function gives the correct output on distribution ${\varvec{Y}}$ and give a bound on the approximation error of the trimming operation.

#### Lemma 7

(Trimmed functions are inaccurate in the positive distribution) If a monotone function $f \in \left\{ 0,1\right\} ^n \rightarrow \left\{ 0,1\right\}$ is trimmed and $f \ne \mathbbm {1}$ (i.e., *f* is not identically 1), then

$$\begin{aligned} {{\,\mathrm{\mathbb {P}}\,}}\left[ f({\varvec{Y}}) = 1 \right] \leqslant \sum _{\ell = 1}^{{c/2}} \left( \frac{k}{n} \right) ^{\ell } \left|{\mathcal M}_\ell (f)\right|. \end{aligned}$$

#### Proof

It suffices to see that, since *f* is trimmed, if $f({\varvec{Y}}) = 1$ and $f \ne \mathbbm {1}$ then there exists a minterm *x* of *f* with Hamming weight between 1 and *c*/2 such that $x \leqslant {\varvec{Y}}$. The result follows from Lemma 3 and the union bound. $\square$

#### Lemma 8

(Approximation by trimming) Let $f \in \left\{ 0,1\right\} ^n \rightarrow \left\{ 0,1\right\}$ be a monotone function, all of whose minterms have Hamming weight at most *c*. We have

$$\begin{aligned} {{\,\mathrm{\mathbb {P}}\,}}\left[ f({\varvec{Y}}) = 1 \text { and } {{\,\mathrm{trim}\,}}(f)({\varvec{Y}}) = 0 \right] \leqslant \sum _{\ell = {c/2}}^{c} \left( \frac{k}{n} \right) ^{\ell } \left|{\mathcal M}_\ell (f)\right|. \end{aligned}$$

#### Proof

If we have $f({\varvec{Y}}) = 1$ and ${{\,\mathrm{trim}\,}}(f)({\varvec{Y}}) = 0$, then there was a minterm *x* of *f* with Hamming weight larger than *c*/2 that was removed by the trimming process. Therefore, since $\left|x\right| \leqslant c$ by assumption, the result follows from Lemma 3 and the union bound. $\square$

### The Approximators

Let ${\mathcal {A}}:= \left\{ {{\,\mathrm{trim}\,}}(\mathrm {cl}(f)) : f : \left\{ 0,1\right\} ^n \rightarrow \left\{ 0,1\right\} \text { is monotone}\right\} .$ Functions in ${\mathcal {A}}$ will be called *approximators*. We define the *approximating* operations $\sqcup , \sqcap : {\mathcal {A}}\times {\mathcal {A}}\rightarrow {\mathcal {A}}$ as follows: for $f, g \in {\mathcal {A}}$, let

$$\begin{aligned} f \sqcup g&:= {{\,\mathrm{trim}\,}}(\mathrm {cl}(f \vee g)), \\ f \sqcap g&:= {{\,\mathrm{trim}\,}}(\mathrm {cl}(f \wedge g)). \end{aligned}$$

We now observe that every input function is an approximator. Indeed, since every input ${\lceil }\left\{ i\right\} {\rceil }$ is closed and trivially trimmed (Lemma 6), we have ${{\,\mathrm{trim}\,}}(\mathrm {cl}({\lceil }\left\{ i\right\} {\rceil })) = {{\,\mathrm{trim}\,}}({\lceil }\left\{ i\right\} {\rceil }) = {\lceil }\left\{ i\right\} {\rceil }$. Thus, ${\lceil }\left\{ i\right\} {\rceil } \in {\mathcal {A}}$ for all $i \in [n]$. Therefore, we can replace each gate of a monotone $\left\{ \vee , \wedge \right\}$-circuit *C* by its corresponding approximating gate, thus obtaining a $\left\{ \sqcup , \sqcap \right\}$-circuit $C^{\mathcal {A}}$ computing an approximator.

The rationale for choosing this set of approximators is as follows. By letting approximators be the trimming of a closed function, we are able to plug the bound on the set of $\ell$-minterms given by the robust sunflower lemma (Lemma 5) on Lemmas 7 and 8 , since the trimming operation can only *reduce* the set of minterms. Moreover, since trimmings can only help to get a negative answer on the negative test distribution, we can safely apply Lemma 4 when bounding the errors of approximation.

### The Lower Bound

In this section, we prove that the function $f_\text {HR}$ requires monotone circuits of size $2^{\varOmega (c)}$. By properly choosing *c* and *k*, this will imply the promised $\exp ({\varOmega (n^{1/2-o(1)})})$ lower bound for the Harnik-Raz function. First, we fix some parameters. Choose *B* as in Lemma 5. Let $T := 18B$. We also let

$$\begin{aligned} k := n^{1/2}, \quad \quad c := \frac{1}{T} \cdot (k/\log n) = \frac{k}{18B \cdot \log n}. \end{aligned}$$

For simplicity, we assume these values are integers. Note that $c = \varTheta (k / \log n) \ll k$.

#### Lemma 9

(Approximators make many errors) For every approximator $f \in {\mathcal {A}}$, we have

$$\begin{aligned} {{\,\mathrm{\mathbb {P}}\,}}[f({\varvec{Y}})=1] + {{\,\mathrm{\mathbb {P}}\,}}[f({\varvec{N}})=0] \leqslant 3/2. \end{aligned}$$

#### Proof

Let $f \in {\mathcal {A}}$. By definition, there exists a closed function *h* such that $f = {{\,\mathrm{trim}\,}}(h)$. Observe that ${\mathcal M}_\ell (f) \subseteq {\mathcal M}_\ell (h)$ for every $\ell \in [c]$. From Lemma 5, we get

$$\begin{aligned} \left|{\mathcal M}_\ell (h)\right| \leqslant (6B c \log n)^\ell = (n/3k)^\ell . \end{aligned}$$

Hence, applying Lemma 7, we obtain that, if $f \ne \mathbbm {1}$, we have

$$\begin{aligned} {{\,\mathrm{\mathbb {P}}\,}}[f({\varvec{Y}})=1] \leqslant \sum _{\ell =1}^{{c/2}} \left( \frac{k}{n} \right) ^{\ell } \left|{\mathcal M}_\ell (h)\right| \leqslant \sum _{\ell =1}^{{c/2}} 3^{-\ell } \leqslant 1/2. \end{aligned}$$

Therefore, for every $f \in {\mathcal {A}}$ we have ${{\,\mathrm{\mathbb {P}}\,}}[f({\varvec{Y}}) = 1] + {{\,\mathrm{\mathbb {P}}\,}}[f({\varvec{N}}) = 0] \leqslant 1 + 1/2 \leqslant 3/2.$

$$\square $$

#### Lemma 10

(*C* is well-approximated by $C^{\mathcal {A}}$) Let *C* be a monotone circuit. We have

$$\begin{aligned} {{\,\mathrm{\mathbb {P}}\,}}[C({\varvec{Y}}) = 1 \text { and } C^{\mathcal {A}}({\varvec{Y}}) = 0] + {{\,\mathrm{\mathbb {P}}\,}}[C({\varvec{N}}) = 0 \text { and } C^{\mathcal {A}}({\varvec{N}}) = 1] \leqslant {{\,\mathrm{size}\,}}(C) \cdot 2^{-\varOmega (c)}. \end{aligned}$$

#### Proof

We begin by bounding the approximation errors under the distribution ${\varvec{Y}}$. We will show that, for two approximators $f,g \in {\mathcal {A}}$, if $f \vee g$ accepts an input from ${\varvec{Y}}$, then $f \sqcup g$ rejects that input with probability at most $2^{-\varOmega (c)}$, and that the same holds for the approximation $f \sqcap g$.

First note that, if $f, g \in {\mathcal {A}}$, then all the minterms of both $f \vee g$ and $f \wedge g$ have Hamming weight at most *c*, since *f* and *g* are trimmed. Let now $h = \mathrm {cl}(f \vee g)$. We have $(f \sqcup g)(x) < (f \vee g)(x)$ only if ${{\,\mathrm{trim}\,}}(h)(x) < h(x)$. Since *h* is closed, we get from Lemma 5 that, for all $\ell \in [c]$, we have

$$\begin{aligned} \left|{\mathcal M}_\ell (h)\right| \leqslant (6B c \log n)^\ell = (n/3k)^\ell . \end{aligned}$$

We then obtain the following inequality by Lemma 8:

$$\begin{aligned} {{\,\mathrm{\mathbb {P}}\,}}\left[ (f \vee g)({\varvec{Y}}) = 1 \text { and } (f \sqcup g)({\varvec{Y}}) = 0 \right] \leqslant \sum _{\ell = {c/2}}^c \left( \frac{k}{n} \right) ^{\ell } \left|{\mathcal M}_\ell (h)\right| \leqslant \sum _{\ell = {c/2}}^c 3^{-\ell } = 2^{-\varOmega (c)}. \end{aligned}$$

The same argument shows ${{\,\mathrm{\mathbb {P}}\,}}\left[ (f \wedge g)({\varvec{Y}}) = 1 \text { and } (f \sqcap g)({\varvec{Y}}) = 0 \right] = 2^{-\varOmega (c)}.$ Since there are ${{\,\mathrm{size}\,}}(C)$ gates in *C*, this implies that ${{\,\mathrm{\mathbb {P}}\,}}[C({\varvec{Y}}) = 1 \text { and } C^{\mathcal {A}}({\varvec{Y}}) = 0] \leqslant {{\,\mathrm{size}\,}}(C) \cdot 2^{-\varOmega (c)}.$

To bound the approximation errors under ${\varvec{N}}$, note that $(f \vee g)(x)=0$ and $(f \sqcup g)(x) = 1$ only if $\mathrm {cl}(f \vee g)(x) \ne (f \vee g)(x)$, since trimming a Boolean function cannot decrease the probability that it rejects an input. Therefore, by Lemma 4 we obtain

$$\begin{aligned} {{\,\mathrm{\mathbb {P}}\,}}\left[ (f \vee g)({\varvec{N}}) = 0 \text { and } (f \sqcup g)({\varvec{N}}) = 1 \right] \leqslant n^{-c} = 2^{-\varOmega (c)}. \end{aligned}$$

The same argument shows ${{\,\mathrm{\mathbb {P}}\,}}\left[ (f \wedge g)({\varvec{N}}) = 0 \text { and } (f \sqcap g)({\varvec{N}}) = 1 \right] = 2^{-\varOmega (c)}.$ Once again, doing this approximation for every gate in *C* allows us to conclude ${{\,\mathrm{\mathbb {P}}\,}}[C({\varvec{N}}) = 0 \text { and } C^{\mathcal {A}}({\varvec{N}}) = 1] \leqslant {{\,\mathrm{size}\,}}(C) \cdot 2^{-\varOmega (c)}.$ This finishes the proof. $\square$

#### Theorem 4

Any monotone circuit computing $f_\text {HR}$ has size $2^{\varOmega (c)} = 2^{\varOmega (n^{1/2}/\log n)}$.

#### Proof

Let *C* be a monotone circuit computing $f_\text {HR}$. Since $k/2 - c \log _2 n = \varOmega (k)$ and $k \ll n$, for large enough *n* we obtain from Lemmas 1 and 2 that

$$\begin{aligned} {{\,\mathrm{\mathbb {P}}\,}}[f_\text {HR}({\varvec{Y}})=1] + {{\,\mathrm{\mathbb {P}}\,}}[f_\text {HR}({\varvec{N}})=0] \geqslant 2-(k-1)/n-2^{-(k/2-c\log _2 n)} \geqslant 9/5. \end{aligned}$$

We then obtain from Lemmas 9 and 10 :

$$\begin{aligned} 9/5&\leqslant {{\,\mathrm{\mathbb {P}}\,}}[f_\text {HR}({\varvec{Y}})=1] + {{\,\mathrm{\mathbb {P}}\,}}[f_\text {HR}({\varvec{N}})=0] \\ {}&\leqslant {{\,\mathrm{\mathbb {P}}\,}}[C({\varvec{Y}}) = 1 \text { and } C^{\mathcal {A}}({\varvec{Y}}) = 0] + {{\,\mathrm{\mathbb {P}}\,}}[C^{\mathcal {A}}({\varvec{Y}})=1] \\ {}&+ {{\,\mathrm{\mathbb {P}}\,}}[C({\varvec{N}}) = 0 \text { and } C^{\mathcal {A}}({\varvec{N}}) = 1] + {{\,\mathrm{\mathbb {P}}\,}}[C^{\mathcal {A}}({\varvec{N}})=0] \\ {}&\leqslant 3/2 + {{\,\mathrm{size}\,}}(C)2^{-\varOmega (c)}. \end{aligned}$$

This implies $${{\,\mathrm{size}\,}}(C) = 2^{\varOmega (c)}$$. $$\square $$

### Are Better Lower Bounds Possible with Robust Sunflowers?

In this section, we allow some degree of imprecision for the sake of brevity and clarity, in order to highlight the main technical ideas of the proof.

A rough outline of how we just proved Theorem 4 is as follows. First, we noted that the minterms of $f_\text {HR}$ are “well-spread”. This is Lemma 3, which states that the probability that a fixed set $A \subseteq [n]$ is contained in a random minterm^4^ of $f_\text {HR}$ is at most $r^{\left|A\right|}$, where $r = k/n$. Moreover, we observed that $f_\text {HR}$ outputs 0 with high probability in a *p*-biased distribution (Lemma 2), where $p=1/2$.

In the rest of the proof, we roughly showed how this implies that DNFs of size approximately $s = c^{c/2}$ and width $w = c/2$ *cannot* approximate $f_\text {HR}$ (Lemma 9).^5^ We also observed that we can approximate the $\vee$ and $\wedge$ of width-*w*, size-*s* DNFs by another width-*w*, size-*s* DNF, bounding the error of approximation by $r^{c/2} \cdot c^{c/2}$. This was proved by noting that conjunctions of width *c*/2 accept a positive input with probability at most $r^{c/2}$, and there are at most $c^{c/2}$ of them. When $c \approx k \approx \sqrt{n}$, we have $(rc)^{c/2} = 2^{-\varOmega (c)}$, and thus *we can* approximate circuits of size $2^{o(c)}$ with width-*w*, size-*s* DNFs (Lemma 10). This yields the lower bound.

There are two essential numerical components in the proof. First, the “spreadness rate” of the function $f_\text {HR}$. A simple counting argument can show that the upper bound of $(k/n)^{\left|A\right|}$ to the probability ${{\,\mathrm{\mathbb {P}}\,}}[x_A \leqslant {\varvec{Y}}]$ is nearly best possible when the support of ${\varvec{Y}}$ is contained in $\left\{ 0,1\right\} ^n_{= k}$ and $k = o(n)$. So this can hardly be improved with the choice of another Boolean function. Secondly, the bounds for the size and width of the DNF approximators come from the robust sunflower lemma (Theorem 3), which was used to employ the approximation method on *p*-biased distributions. Since the bound of Theorem 3 is essentially best possible as well, as observed in [3], we cannot hope to get better approximation bounds on a *p*-biased distribution from sunflowers. Therefore, there does not seem to be much room for getting better lower bounds for monotone circuits using the classical approximation method with sunflowers, if we use *p*-biased distributions. To get beyond $2^{\varOmega (\sqrt{n})}$, another approach seems to be required.

## Lower Bound for $\mathsf {Clique}_{k,n}$

Recall that the Boolean function $$\mathsf {Clique}_{k,n}: \{0,1\}^{\left( {\begin{array}{c}n\\ 2\end{array}}\right) } \rightarrow \{0,1\}$$ receives a graph on *n* vertices as an input and outputs a 1 if this graph contains a clique on *k* vertices. In this section, we prove an $n^{\varOmega (\delta ^2 k)}$ lower bound on the monotone circuit size of $\mathsf {Clique}_{k,n}$ for $k \leqslant n^{(1/3)-\delta }$.

We note that the first superpolynomial lower bound for the monotone circuit complexity of $\mathsf {Clique}_{k,n}$ was given by Razborov [23], who proved a $n^{\varOmega (k)}$ lower bound for $k \leqslant \log n$. Soon after, Alon and Boppana [2] proved a $n^{\varOmega (\sqrt{k})}$ for $\mathsf {Clique}_{k,n}$ when $k \leqslant n^{2/3 - o(1)}$. This exponential lower bound was better than Razborov’s, as it could be applied to a larger range of *k*, but it was short of the obvious upper bound of $n^{O(k)}$. Our result finally closes that gap, by proving that the monotone complexity of $\mathsf {Clique}_{k,n}$ is $n^{\varTheta (k)}$ even for large *k*.

As in Sect. 2, we will follow the approximation method. However, instead of using sunflowers as in [2, 23] or robust sunflowers as in [24], we introduce a notion of *clique-sunflowers* and employ it to bound the errors of approximation.

### Notation for this Section

In this section, we will often refer to graphs on *n* vertices and Boolean strings in $$\left\{ 0,1\right\} ^{\left( {\begin{array}{c}n\\ 2\end{array}}\right) }$$ interchangeably. For $A \subseteq [n]$, let $K_A$ be the graph on *n* vertices with a clique on *A* and no other edges. When $\left|A\right| \leqslant 1$, the graph $K_A$ is the empty graph with *n* vertices and 0 edges (corresponding to the Boolean string all of which $$\left( {\begin{array}{c}n\\ 2\end{array}}\right) $$ entries are equal to 0.) The *size* of $K_A$ is $\left|A\right|$. Let also $${\lceil }A{\rceil } : \left\{ 0,1\right\} ^{\left( {\begin{array}{c}n\\ 2\end{array}}\right) } \rightarrow \left\{ 0,1\right\} $$ denote the indicator function of containing $K_A$, which satisfies

$$\begin{aligned} {\lceil }A{\rceil }(G) = 1 \iff K_A \subseteq G. \end{aligned}$$

Functions of the forms ${\lceil }A{\rceil }$ are called *clique-indicators.* Moreover, if $\left|A\right| = \ell$, we say that ${\lceil }A{\rceil }$ is a clique-indicator of *size* equal to $\ell$. When $\left|A\right| \leqslant 1$, the function ${\lceil }A{\rceil }$ is the constant function $\mathbbm {1}$.

For $p \in (0,1)$, we denote by ${\varvec{G}}_{n,p}$ the Erdős-Rényi random graph, a random graph on *n* vertices in which each edge appears independently with probability *p*.

Let $$f : \left\{ 0,1\right\} ^{\left( {\begin{array}{c}n\\ 2\end{array}}\right) } \rightarrow \left\{ 0,1\right\} $$ be monotone and suppose $\ell \in \left\{ 1,\dots , \delta k\right\}$. We define

$$\begin{aligned} {\mathcal M}_\ell (f) :=\{A \in \textstyle \left( {\begin{array}{c}[n]\\ \ell \end{array}}\right) : f(K_A) = 1 \text { and } f(K_{A\setminus \{a\}}) = 0\text { for all } a \in A\}. \end{aligned}$$

Elements of ${\mathcal M}_\ell (f)$ are called $\ell$-*clique-minterms* of *f*.

### Clique-Sunflowers

Here we introduce the notion of *clique-sunflowers*, which is analogous to that of robust sunflowers for “clique-shaped” set systems.

#### Definition 5

*(Clique-sunflowers)* Let $\varepsilon , p \in (0,1)$. Let ${\mathcal S}$ be a family of subsets of [*n*] and let $Y := \bigcap {\mathcal S}$. The family ${\mathcal S}$ is called a $(p, \varepsilon )$-*clique-sunflower* if

$$\begin{aligned} {{\,\mathrm{\mathbb {P}}\,}}\left[ \exists A \in S: K_A \subseteq {\varvec{G}}_{n,p} \cup K_Y \right] > 1-\varepsilon . \end{aligned}$$

Equivalently, the family ${\mathcal S}$ is a clique-sunflower if the family $$\left\{ K_A : A \in {\mathcal S}\right\} \subseteq \left( {\begin{array}{c}[n]\\ 2\end{array}}\right) $$ is a $(p,\varepsilon )$-robust sunflower, since $K_A \cap K_B = K_{A \cap B}$.

Though clique-sunflowers may seem similar to regular sunflowers, the importance of this definition is that it allows us to explore the “clique-shaped” structure of the sets of the family, and thus obtain an asymptotically better upper bound on the size of sets that do not contain a clique-sunflower.

#### Lemma 11

(Clique-sunflower lemma) Let $\varepsilon < e^{-1/2}$ and let $${\mathcal S}\subseteq \left( {\begin{array}{c}[n]\\ \ell \end{array}}\right) $$. If the family ${\mathcal S}$ satisfies $$\left|{\mathcal S}\right| > \ell !(2\ln (1/\varepsilon ))^\ell (1/p)^{\left( {\begin{array}{c}\ell \\ 2\end{array}}\right) }$$, then ${\mathcal S}$ contains a $(p,\varepsilon )$-clique-sunflower.

Observe that, whereas the bounds for “standard” robust sunflowers (Theorems 2 and 3) would give us an exponent of $$\left( {\begin{array}{c}\ell \\ 2\end{array}}\right) $$ on the $\log (1/\varepsilon )$ factor, Lemma 11 give us only an $\ell$ at the exponent. As we shall see, this is asymptotically better for our choice of parameters.

We defer the proof of Lemma 11 to Sect. 3.8. The proof is based on an application of Janson’s inequality [13], as in the original robust sunflower lemma of [24] (Theorem 2).

### Test Distributions

We now define the positive and negative test distributions. First, we fix some parameters that will be used throughout the proof. Fix $\delta \in (0,1/3)$. Let

2 $$\begin{aligned} k = n^{1/3-\delta } \quad \text { and }\quad p := n^{-2/(k-1)}. \end{aligned}$$

For simplicity, we will assume from now on that $\delta k$ and $\delta k/2$ are integers.

#### Remark 5

(Parameters are now fixed) From now on until the end of Sect. 3.7, the symbols $p, \delta$ and *k* refer to fixed parameters, and will always unambiguously refer to the values just fixed. This will only change in Sect. 3.8, which is independent of the proof of the lower bound for $\mathsf {Clique}_{k,n}$, and in which we will permit ourselves to reuse some of these symbols for other purposes. This means that, whenever $p, \delta$ and *k* appear in the following discussion, the reader must bear in mind that $p=n^{-2/(k-1)}$, $\delta$ is a fixed number inside (0, 1/3) and *k* is fixed to be $k=n^{1/3-\delta }$.

We observe that the probability that ${\varvec{G}}_{n,p}$ has a *k*-clique is bounded away from 1.

#### Lemma 12

We have $${{\,\mathrm{\mathbb {P}}\,}}[\ {\varvec{G}}_{n,p} \text { contains a k-clique}\ ] \leqslant 3/4$$.

#### Proof

There are $$\left( {\begin{array}{c}n\\ k\end{array}}\right) \leqslant (en/k)^k$$ potential *k*-cliques, each present in ${\varvec{G}}_{n,p}$ with probability $$p^{\left( {\begin{array}{c}k\\ 2\end{array}}\right) } = n^{-k}$$. By a union bound, we have ${{\,\mathrm{\mathbb {P}}\,}}[\ {\varvec{G}}_{n,p} \text { contains a k-clique}\ ] \leqslant (e/k)^k \leqslant (e/3)^3 \leqslant 3/4$. $\square$

#### Definition 6

Let ${\varvec{Y}}$ be the uniform random graph chosen from all possible $K_A$, where $\left|A\right| = k$. In other words, the distribution ${\varvec{Y}}$ samples a random minterm of $\mathsf {Clique}_{k,n}$. We call ${\varvec{Y}}$ the *positive test distribution*. Let also ${\varvec{N}}:= {\varvec{G}}_{n,p}$. We call ${\varvec{N}}$ the *negative test distribution*.

From Lemma 12, we easily obtain the following corollary.

#### Corollary 1

We have $$ {{\,\mathrm{\mathbb {P}}\,}}[\mathsf {Clique}_{k,n}({\varvec{Y}})=1] + {{\,\mathrm{\mathbb {P}}\,}}[\mathsf {Clique}_{k,n}({\varvec{N}})=0] \geqslant 5/4. $$

We now prove an analogous result to that of Lemma 3, which shows that the positive distribution ${\varvec{Y}}$ is unlikely to contain a large fixed clique.

#### Lemma 13

For every $\ell \leqslant k$ and $A \subseteq [n]$ such that $\left|A\right|=\ell$, we have

$$\begin{aligned} {{\,\mathrm{\mathbb {P}}\,}}[K_A \leqslant {\varvec{Y}}] \leqslant \left( k/n \right) ^{\ell }. \end{aligned}$$

#### Proof

The distribution ${\varvec{Y}}$ samples a set ${\varvec{B}}$ uniformly at random from $$\left( {\begin{array}{c}[n]\\ k\end{array}}\right) $$ and returns the graph $K_{{\varvec{B}}}$. Note that $K_A \subseteq K_{{\varvec{B}}}$ if and only if $A \subseteq {\varvec{B}}$. We have

$$\begin{aligned} {{\,\mathrm{\mathbb {P}}\,}}[K_A \leqslant {\varvec{Y}}] = {{\,\mathrm{\mathbb {P}}\,}}[A \subseteq {\varvec{B}}] = \frac{\left( {\begin{array}{c}n-k\\ k-\ell \end{array}}\right) }{\left( {\begin{array}{c}n\\ k\end{array}}\right) } \leqslant \left( \frac{k}{n} \right) ^{\ell }. \end{aligned}$$

$$\square $$

### A Closure Operator

As in Sect. 2.4, we define here a closure operator in the lattice of monotone Boolean functions. We will again prove that the closure of a function will be a good approximation for it on the negative test distribution. However, unlike Sect. 2.4, instead of bounding the set of minterms, we will bound the set of “clique-shaped” minterms, as we shall see. Finally, we will observe that input functions are also closed. Henceforth, we fix the error parameter

3 $$\begin{aligned} \varepsilon := n^{-k}. \end{aligned}$$

#### Definition 7

*(Closed functions)* We say that $$f \in \left\{ 0,1\right\} ^{\left( {\begin{array}{c}n\\ 2\end{array}}\right) } \rightarrow \left\{ 0,1\right\} $$ is *closed* if, for every $A \subseteq [n]$ such that $\left|A\right| \in \left\{ 2, \dots , \delta k\right\}$, we have

$$\begin{aligned} {{\,\mathrm{\mathbb {P}}\,}}[\ f({\varvec{N}}\vee K_A) = 1\ ] > 1 - \varepsilon \ \Longrightarrow \ f(K_A) = 1. \end{aligned}$$

#### Remark 6

(On the parametrization of closedness) Similarly to the Harnik-Raz case (see Remark 2), the definition of a *closed* function depends on three parameters: the probability *p*, which controls the distribution ${\varvec{N}}$ (as discussed in Definition 6), the parameter $\varepsilon$, defined in (3), and the parameter *k*. Since all of these three parameters are fixed until the end of Sect. 3.7 (see Remark 5), and no other reference to closed functions will be made after that, it is safe to omit them without risk of confusion. Therefore, we will henceforth say that some function is *closed* without any further specification about the parameters. However, the reader must bear in mind that, whenever a function is said to be *closed*, the *fixed* parameters $p, \varepsilon$ and *k* are in view.

#### Remark 7

(Definitions of closedness compared) Definition 8 bears great resemblance to Definition 3, which also talks about a notion of *closed monotone functions* in the context of lower bounds for the function of Harnik and Raz. Apart from the different parametrizations, the main difference between those two definitions is that, whereas Definition 3 looks into *all* inputs of Hamming weight at most *c*, here we only care about *clique-shaped* inputs of size at most $\delta k$.

As before, we can define the closure of a monotone Boolean function *f*.

#### Definition 8

*(Closure operator)* Let *f* be a monotone Boolean function. We denote by $\mathrm {cl}(f)$ the unique minimal closed monotone Boolean function such that $f \leqslant \mathrm {cl}(f)$.

#### Remark 8

(On closure) We note again that $\mathrm {cl}(f)$ is well-defined (the same arguments of Remark 3 apply here) and remark that its definition also depends on the parameters $p, \varepsilon$ and *k* (see Remark 6), which are fixed throughout the proof, and therefore can be safely omitted.

#### Lemma 14

(Approximation by closure) For every monotone $$f : \left\{ 0,1\right\} ^{\left( {\begin{array}{c}n\\ 2\end{array}}\right) } \rightarrow \left\{ 0,1\right\} $$, we have

$$\begin{aligned} {{\,\mathrm{\mathbb {P}}\,}}\left[ f({\varvec{N}}) = 0 \text { and } \mathrm {cl}(f)({\varvec{N}}) = 1 \right] \leqslant n^{-(2/3)k}. \end{aligned}$$

#### Proof

We repeat the same argument as that of Lemma 4. Since there are at most $n^{\delta k}$ graphs $K_A$ such that $\left|A\right| \leqslant \delta k$ and $\varepsilon = n^{-k}$, the final bound then becomes $n^{-k} \cdot n^{\delta k} \leqslant n^{-(2/3)k}$. $\square$

By employing the clique-sunflower lemma (Lemma 11), we are able to bound the set of $\ell$-clique-minterms of closed monotone functions.

#### Lemma 15

(Closed functions have few minterms) If a monotone function $$f : \left\{ 0,1\right\} ^{\left( {\begin{array}{c}n\\ 2\end{array}}\right) } \rightarrow \left\{ 0,1\right\} $$ is closed, then, for all $\ell \in \left\{ 2,\dots , \delta k\right\}$, we have

$$\begin{aligned} \left|{\mathcal M}_\ell (f)\right|&\leqslant n^{2\ell /3}. \end{aligned}$$

#### Proof

Recall that $p = n^{-2/(k-1)}$ and $\varepsilon = n^{-k}$ (see (2) and (3)). Applying the same strategy of Lemma 5, replacing the application of Theorem 3 (robust sunflower theorem) by Lemma 11 (clique-sunflower lemma), we obtain

$$\begin{aligned} \left|{\mathcal M}_\ell (f)\right|&\leqslant \ell !(2\log (1/\varepsilon ))^\ell (1/p)^{\left( {\begin{array}{c}\ell \\ 2\end{array}}\right) } \leqslant (2\ell k \log n)^\ell \cdot {p^{-\left( {\begin{array}{c}\ell \\ 2\end{array}}\right) }} \\ {}&\leqslant (2\delta k^2 \log n)^\ell \cdot {n^{2\left( {\begin{array}{c}\ell \\ 2\end{array}}\right) /(k-1)}} \leqslant (n^{2/3-2\delta } \log n)^\ell \cdot n^{\delta \ell } \leqslant n^{2\ell /3}. \end{aligned}$$

$$\square $$

#### Lemma 16

(Input functions are closed) Let $i,j \in [n]$ be such that $i \ne j$. For large enough *n*, the Boolean function ${\lceil }\left\{ i,j\right\} {\rceil }$ is closed.

#### Proof

Fix $i,j \in [n]$ such that $i \ne j$. Let $A \subseteq [n]$ be such that $\left|A\right| \leqslant \delta k$ and suppose that ${\lceil }\left\{ i,j\right\} {\rceil }(K_A) = 0$. Note that ${\lceil }\left\{ i,j\right\} {\rceil }(K_A) = 0$ is equivalent to $\left\{ i,j\right\} \not \subseteq A$. This implies that $\left\{ i,j\right\}$ is an edge of ${\varvec{{\varvec{N}}}}\cup K_A$ if and only if $\left\{ i,j\right\}$ is an edge of ${\varvec{N}}$. Therefore, we have

$$\begin{aligned} {{\,\mathrm{\mathbb {P}}\,}}[{\lceil }\left\{ i,j\right\} {\rceil }({\varvec{N}}\vee K_A)=1]&= {{\,\mathrm{\mathbb {P}}\,}}[{\lceil }\left\{ i,j\right\} {\rceil }({\varvec{N}})=1] \\ {}&= {{\,\mathrm{\mathbb {P}}\,}}[\left\{ i,j\right\} \text { is an edge of } {\varvec{G}}_{n,p}] \\ {}&= n^{-2/(k-1)}, \end{aligned}$$

since ${\varvec{N}}= {\varvec{G}}_{n,p}$ and $p = n^{-2/(k-1)}$ (see (2), Remark 5 and Definiton 6). It now suffices to show that, for large enough *n*, we have $p \leqslant 1-\varepsilon =1-n^{-k}$ (recall from (3) that $\varepsilon = n^{-k}$).

For convenience, let $\alpha = 1/3-\delta$. Note that $k=n^{\alpha }$. For large enough *n*, we have

$$\begin{aligned} \frac{2 \cdot \log n}{n^{\alpha }-1} \geqslant n^{-n^\alpha } + n^{-2n^\alpha }. \end{aligned}$$

Using the inequality $\log (1-x) \geqslant -x -x^2$ for $x \in [0,1/2]$, we get

$$\begin{aligned} \frac{2 \cdot \log n}{k-1} = \frac{2 \cdot \log n}{n^{\alpha }-1} \geqslant n^{-n^\alpha } + n^{-2n^\alpha } \geqslant -\log (1-n^{-n^\alpha }) = -\log (1-n^{-k}). \end{aligned}$$

Therefore, we have

$$\begin{aligned} n^{-2/(k-1)} \leqslant 1-n^{-k}, \end{aligned}$$

and we conclude that ${\lceil }\left\{ i,j\right\} {\rceil }$ is closed. $\square$

### Trimmed Monotone Functions

In this section, we define again a trimming operation for Boolean functions and prove analogous bounds to that of Sect. 2.5.

#### Definition 9

*(Clique-shaped and trimmed functions)* We say that a function $$f : \left\{ 0,1\right\} ^{\left( {\begin{array}{c}n\\ 2\end{array}}\right) } \rightarrow \left\{ 0,1\right\} $$ is *clique-shaped* if, for every minterm *x* of *f*, there exists $A \subseteq [n]$ such that $x = K_A$. Moreover, we say that *f* is *trimmed* if *f* is clique-shaped and all the clique-minterms of *f* have size at most $\delta k/2$. For a clique-shaped function *f*, we define the trimming operation ${{\,\mathrm{trim}\,}}(f)$ as follows:

$$\begin{aligned} {{\,\mathrm{trim}\,}}(f) := \bigvee _{\ell = 1}^{\delta k/2} \bigvee _{A \in {\mathcal M}_{\ell }(f)} {\lceil }A{\rceil }. \end{aligned}$$

That is, the ${{\,\mathrm{trim}\,}}$ operation takes out from *f* all the clique-indicators of size larger than $\delta k/2$, yielding a trimmed function.

#### Remark 9

(Parametrization of ${{\,\mathrm{trim}\,}}(\cdot )$ and other remarks) Analogously to the Harnik-Raz case (see Remark 4), the definition of trimmed functions depends on the choice of the parameters $\delta$ and *k*. As these parameters are fixed (see Remark 5), the operator ${{\,\mathrm{trim}\,}}(\cdot )$ is well-defined. Moreover, if all clique-minterms of *f* have size larger than $\delta k/2$ (i.e., if ${\mathcal M}_{\ell }(f) = \emptyset$ for all $\ell \in [\delta k/2]$), then ${{\,\mathrm{trim}\,}}(f)$ is the constant function that outputs 0. Finally, if *f* is the constant function $\mathbbm {1}$, then ${{\,\mathrm{trim}\,}}(f) = \mathbbm {1}$, because $\mathbbm {1}$ contains a clique-minterm of size equal to 1 (a clique containing one vertex and no edges).

Imitating the proofs of Lemmas 7 and 8 , replacing Lemma 3 by Lemma 13, we may now obtain the following lemmas.

#### Lemma 17

(Trimmed functions are inaccurate in the positive distribution) If a monotone function $$f : \left\{ 0,1\right\} ^{\left( {\begin{array}{c}n\\ 2\end{array}}\right) } \rightarrow \left\{ 0,1\right\} $$ is a trimmed clique-shaped function such that $f \ne \mathbbm {1}$, then

$$\begin{aligned} {{\,\mathrm{\mathbb {P}}\,}}\left[ f({\varvec{Y}}) = 1 \right] \leqslant \sum _{\ell = 2}^{\delta k/2} \left( \frac{k}{n} \right) ^{\ell } \left|{\mathcal M}_\ell (f)\right|. \end{aligned}$$

#### Lemma 18

(Approximation by trimming) Let $$f : \left\{ 0,1\right\} ^{\left( {\begin{array}{c}n\\ 2\end{array}}\right) } \rightarrow \left\{ 0,1\right\} $$ be a clique-shaped monotone function, all of whose clique-minterms have size at most $\delta k$. We have

$$\begin{aligned} {{\,\mathrm{\mathbb {P}}\,}}\left[ f({\varvec{Y}}) = 1 \text { and } {{\,\mathrm{trim}\,}}(f)({\varvec{Y}}) = 0 \right] \leqslant \sum _{\ell ={\delta k/2}}^{\delta k} \left( \frac{k}{n} \right) ^{\ell } \left|{\mathcal M}_\ell (f)\right|. \end{aligned}$$

### Approximators

Similarly as in Sect. 2.6, we will consider a set of *approximators* ${\mathcal {A}}$. Let

$$\begin{aligned} {\mathcal {A}}:= \{{{\,\mathrm{trim}\,}}(\mathrm {cl}(f)) : f \in \left\{ 0,1\right\} ^{\left( {\begin{array}{c}n\\ 2\end{array}}\right) } \rightarrow \left\{ 0,1\right\} \text { is monotone and clique-shaped}\}. \end{aligned}$$

Functions in ${\mathcal {A}}$ are called *approximators*. Note that every function in ${\mathcal {A}}$ is clique-shaped and is the trimming of a closed function. Moreover, observe that every edge-indicator ${\lceil }\left\{ u,v\right\} {\rceil }$ belongs to ${\mathcal {A}}$, since every edge-indicator is closed by Lemma 16.

Let $f, g \in {\mathcal {A}}$ such that $f = \bigvee _{i=1}^t {\lceil }A_i{\rceil }$ and $g = \bigvee _{j=1}^s {\lceil }B_j{\rceil }$. We define $\bigwedge (f,g) := \bigvee _{i=1}^t \bigvee _{j=1}^s {\lceil }A_i \cup B_j{\rceil }$. We also define operations $\sqcup , \sqcap : {\mathcal {A}}\times {\mathcal {A}}\rightarrow {\mathcal {A}}$ as follows:

$$\begin{aligned} f \sqcup g&:= {{\,\mathrm{trim}\,}}(\mathrm {cl}(f \vee g)), \\ f \sqcap g&:= {{\,\mathrm{trim}\,}}\left( \mathrm {cl}\left( \bigwedge (f,g) \right) \right) . \end{aligned}$$

It’s easy to see that, if $f,g \in {\mathcal {A}}$, then $f \sqcup g \in {\mathcal {A}}$. To see that $f \sqcap g \in {\mathcal {A}}$, note that $\bigwedge (f,g)$ is also a monotone clique-shaped function.

#### Remark 10

(Reason for definition of $\sqcap$) The reason for defining $\sqcap$ in that way is as follows. First observe that $f \wedge g = \bigvee _{i = 1}^t \bigvee _{j=1}^s ({\lceil }A_i{\rceil } \wedge {\lceil }B_j{\rceil })$. We simply replace each ${\lceil }A_i{\rceil } \cap {\lceil }B_j{\rceil }$ with ${\lceil }A_i \cup B_j{\rceil }$, thus obtaining $f \sqcap g$. In general, since ${\lceil }A_i \cup B_j{\rceil }$ is a larger conjunction than ${\lceil }A_i{\rceil } \wedge {\lceil }B_j{\rceil }$, we have $\bigwedge (f,g) \leqslant f \wedge g$. However, note that, for every $A \subseteq [n]$, we have $\bigwedge (f,g)(K_A) = (f \wedge g)(K_A)$. Thus, the transformation from $f \wedge g$ to $\bigwedge (f,g)$ incurs no mistakes in the positive distribution ${\varvec{Y}}$.

If *C* is a monotone $\left\{ \vee , \wedge \right\}$-circuit, let $C^{\mathcal {A}}$ be the corresponding $\left\{ \sqcup , \sqcap \right\}$-circuit, obtained by replacing each $\vee$-gate by a $\sqcup$-gate, and each $\wedge$-gate by an $\sqcap$-gate. Note that $C^{\mathcal {A}}$ computes an approximator.

### The Lower Bound

In this section we obtain the lower bound for the clique function. Recall that $k = n^{1/3-\delta }$. We will prove that the monotone complexity of $\mathsf {Clique}_{k,n}$ is $n^{\varOmega (\delta ^2 k)}$.

Repeating the same arguments of Lemmas 9 and 10 , we obtain the following analogous lemmas.

#### Lemma 19

(Approximators make many errors) For every $f \in {\mathcal {A}}$, we have

$$\begin{aligned} {{\,\mathrm{\mathbb {P}}\,}}[f({\varvec{Y}})=1] + {{\,\mathrm{\mathbb {P}}\,}}[f({\varvec{N}})=0] \leqslant 1+o(1). \end{aligned}$$

#### Proof

Let $f \in {\mathcal {A}}$. By definition, there exists a closed function *h* such that $f = {{\,\mathrm{trim}\,}}(h)$. Observe that ${\mathcal M}_\ell (f) \subseteq {\mathcal M}_\ell (h)$ for every $\ell \in \left\{ 2,\dots ,\delta k/2\right\}$. By Lemmas 15 and 17 , if $f \in {\mathcal {A}}$ is such that $f \ne \mathbbm {1}$, then

$$\begin{aligned} {{\,\mathrm{\mathbb {P}}\,}}[f({\varvec{Y}})=1] \leqslant \sum _{\ell =2}^{\delta k/2} \left( \frac{k}{n} \right) ^{\ell } \left|{\mathcal M}_\ell (h)\right| \leqslant \sum _{\ell =2}^{\delta k/2} \left( \frac{k}{n^{1/3}}\right) ^\ell \leqslant \sum _{\ell =2}^{\delta k/2} n^{-\delta \ell } = o(1). \end{aligned}$$

Therefore, for every $f \in {\mathcal {A}}$ we have ${{\,\mathrm{\mathbb {P}}\,}}[f({\varvec{Y}}) = 1] + {{\,\mathrm{\mathbb {P}}\,}}[f({\varvec{N}}) = 0] \leqslant 1 + o(1).$ $\square$

#### Lemma 20

(*C* is well-approximated by $C^{\mathcal {A}}$) Let *C* be a monotone circuit. We have

$$\begin{aligned} {{\,\mathrm{\mathbb {P}}\,}}[C({\varvec{Y}}) = 1 \text { and } C^{\mathcal {A}}({\varvec{Y}}) = 0] + {{\,\mathrm{\mathbb {P}}\,}}[C({\varvec{N}}) = 0 \text { and } C^{\mathcal {A}}({\varvec{N}}) = 1] \leqslant {{\,\mathrm{size}\,}}(C)\cdot O(n^{-\delta ^2 k / 2}). \end{aligned}$$

#### Proof

To bound the approximation errors under the distribution ${\varvec{Y}}$, first note that, if $f, g \in {\mathcal {A}}$, then all the clique-minterms of both $f \vee g$ and $f \wedge g$ have size at most $\delta k$. Moreover, if $(f \vee g)(x)=1$ but $(f \sqcup g)(x) = 0$, then ${{\,\mathrm{trim}\,}}(\mathrm {cl}(f \vee g)(x)) \ne \mathrm {cl}(f \vee g)(x)$. Therefore, we obtain by Lemmas 15 and 18 that, for $f, g \in {\mathcal {A}}$, we have

$$\begin{aligned} {{\,\mathrm{\mathbb {P}}\,}}\left[ (f \vee g)({\varvec{Y}}) = 1 \text { and } (f \sqcup g)({\varvec{Y}}) = 0 \right]&\leqslant \sum _{\ell = \delta k /2}^{\delta k} \left( \frac{k}{n} \right) ^{\ell } \left|{\mathcal M}_\ell (\mathrm {cl}(f \vee g))\right| \\ {}&\leqslant \sum _{\ell = \delta k /2}^{\delta k} n^{-\delta \ell } = O(n^{-\delta ^2 k / 2}). \end{aligned}$$

As observed in Remark 10, we have $\bigwedge (f,g)({\varvec{Y}}) = (f \wedge g)({\varvec{Y}})$. Thus, once again, the only approximation mistakes incurred by changing a $\wedge$-gate for a $\sqcap$-gate comes from the trimming operation. Again, we conclude

$$\begin{aligned} {{\,\mathrm{\mathbb {P}}\,}}\left[ (f \wedge g)({\varvec{Y}}) = 1 \text { and } (f \sqcap g)({\varvec{Y}}) = 0 \right] = O(n^{-\delta ^2 k / 2}), \end{aligned}$$

which implies

$$\begin{aligned} {{\,\mathrm{\mathbb {P}}\,}}[C({\varvec{Y}}) = 1 \text { and } C^{\mathcal {A}}({\varvec{Y}}) = 0] \leqslant {{\,\mathrm{size}\,}}(C) \cdot O(n^{-\delta ^2 k / 2}). \end{aligned}$$

Similarly, to bound the approximation errors under ${\varvec{N}}$, note that $(f \vee g)(x)=0$ and $(f \sqcup g)(x) = 1$ only if $\mathrm {cl}(f \vee g)(x) \ne (f \vee g)(x)$. Therefore, we obtain by Lemma 14 that, for $f, g \in {\mathcal {A}}$, we have

$$\begin{aligned} {{\,\mathrm{\mathbb {P}}\,}}\left[ (f \vee g)({\varvec{N}}) = 0 \text { and } (f \sqcup g)({\varvec{N}}) = 1 \right] \leqslant n^{-(2/3)k}. \end{aligned}$$

Moreover, note that $\bigwedge (f,g) \leqslant f \wedge g$. As $f \sqcap g = {{\,\mathrm{trim}\,}}(\mathrm {cl}(\bigwedge (f,g)))$, we obtain that $(f \wedge g)(x)=0$ and $(f \sqcap g)(x) = 1$ only if $\mathrm {cl}(\bigwedge (f,g))(x) > \bigwedge (f,g)(x)$. Therefore, we also have

$$\begin{aligned} {{\,\mathrm{\mathbb {P}}\,}}\left[ (f \wedge g)({\varvec{N}}) = 0 \text { and } (f \sqcap g)({\varvec{N}}) = 1 \right] \leqslant n^{-(2/3)k}. \end{aligned}$$

By the union bound, we conclude:

$$\begin{aligned} {{\,\mathrm{\mathbb {P}}\,}}[C({\varvec{N}}) = 0 \text { and } C^{\mathcal {A}}({\varvec{N}}) = 1] \leqslant {{\,\mathrm{size}\,}}(C)\cdot n^{-(2/3)k}. \end{aligned}$$

This finishes the proof. $\square$

We now prove the lower bound for the clique function.

#### Theorem 5

Let $\delta \in (0,1/3)$ and $k=n^{1/3-\delta }$. The monotone circuit complexity of $\mathsf {Clique}_{k,n}$ is $\varOmega (n^{\delta ^2 k/2})$.

#### Proof

Let *C* be a monotone circuit computing $\mathsf {Clique}_{k,n}$. For large *n*, we obtain from Corollary 1 and Lemmas 19 and 20

$$\begin{aligned} 5/4&\leqslant {{\,\mathrm{\mathbb {P}}\,}}[\mathsf {Clique}_{k,n}({\varvec{Y}})] + {{\,\mathrm{\mathbb {P}}\,}}[\mathsf {Clique}_{k,n}({\varvec{N}})] \\ {}&\leqslant {{\,\mathrm{\mathbb {P}}\,}}[C({\varvec{Y}}) = 1 \text { and } C^{\mathcal {A}}({\varvec{Y}}) = 0] + {{\,\mathrm{\mathbb {P}}\,}}[C^{\mathcal {A}}({\varvec{Y}})=1] \\ {}&\quad + {{\,\mathrm{\mathbb {P}}\,}}[C({\varvec{N}}) = 0 \text { and } C^{\mathcal {A}}({\varvec{N}}) = 1] + {{\,\mathrm{\mathbb {P}}\,}}[C^{\mathcal {A}}({\varvec{N}})=1] \\ {}&\leqslant 1 + o(1) + {{\,\mathrm{size}\,}}(C) \cdot O(n^{-\delta ^2 k / 2}). \end{aligned}$$

This implies $${{\,\mathrm{size}\,}}(C) = \varOmega (n^{\delta ^2 k/2})$$. $$\square $$

### Proof of Lemma 11 (Clique-Sunflowers)

In this section, we give the proof of Lemma 11. The proof is essentially the same as the one given by Rossman for Theorem 2 in [24]. We will rely on an inequality due to Janson [13] (see also Theorem 2.18 in [14]).

#### Lemma 21

(Janson’s inequality [13]) Let ${\mathcal {F}}$ be a nonempty hypergraph on [*n*] and let ${\varvec{W}}\subseteq _p [n]$. Define $\mu$ and $\varDelta$ in the following way:

$$\begin{aligned} \mu&:= \quad \;\; \sum _{F \in {\mathcal {F}}} \quad \;\; {{\,\mathrm{\mathbb {P}}\,}}[F \subseteq {\varvec{W}}], \\ \varDelta&:= \sum _{\begin{array}{c} F, H \in {\mathcal {F}} \\ F \cap H \ne \emptyset \end{array}} {{\,\mathrm{\mathbb {P}}\,}}[F \cup H \subseteq {\varvec{W}}]. \end{aligned}$$

Then we have

$$\begin{aligned} {{\,\mathrm{\mathbb {P}}\,}}[\forall F \in {\mathcal {F}} : F \not \subseteq {\varvec{W}}] \leqslant \exp \{-\mu ^2/\varDelta \}. \end{aligned}$$

The following estimates appear in an unpublished note due to Rossman [25], and a slightly weaker form appears implicitly in [24]. We reproduce the proof for completeness.

#### Lemma 22

(Lemma 8 of [25]) Let $s_0(t), s_1(t), \dots$ be the sequence of polynomials defined by

$$\begin{aligned} s_0(t) := 1 \quad \text {and}\quad s_\ell (t) := t \sum _{j=0}^{\ell -1} \left( {\begin{array}{c}\ell \\ j\end{array}}\right) s_j(t). \end{aligned}$$

For all $t > 0$, we have $s_\ell (t) \leqslant \ell !(t+1/2)^\ell .$

#### Proof

We first prove by induction on $\ell$ that $s_\ell (t) \leqslant \ell !(\log (1/t+1))^{-\ell }$, as follows:

$$\begin{aligned} s_\ell (t) = t \sum _{j=0}^{\ell -1} \left( {\begin{array}{c}\ell \\ j\end{array}}\right) s_j(t)&\leqslant t \sum _{j=0}^{\ell -1} \left( {\begin{array}{c}\ell \\ j\end{array}}\right) j!(\log (1/t+1))^{-j} \\ {}&= t \ell !(\log (1/t+1))^{-\ell } \sum _{j=0}^{\ell -1} \frac{(\log (1/t+1))^{\ell -j}}{(\ell -j)!} \\ {}&\leqslant t \ell !(\log (1/t+1))^{-\ell } \left( -1+ \sum _{j=0}^{\infty } \frac{(\log (1/t+1))^{j}}{j!} \right) \\ {}&= t\ell !(\log (1/t+1))^{-\ell } (-1+\exp (\log (1/t+1))) \\ {}&= \ell !(\log (1/t+1))^{-\ell }. \end{aligned}$$

To conclude the proof, we apply the inequality $1/\log (1/t+1) < t+1/2$ for all $t > 0$.

$$\square $$

We will also need the following auxiliary definition.

#### Definition 10

Let $\varepsilon ,p,q \in (0,1)$. Let ${\varvec{U}}_{n,q} \subseteq [n]$ be a *q*-random subset of [*n*] independent of ${\varvec{G}}_{n,p}$. Let ${\mathcal S}$ be a family of subsets of [*n*] and let $B := \bigcap {\mathcal S}$. The family ${\mathcal S}$ is called a $(p, q, \varepsilon )$-*clique-sunflower* if

$$\begin{aligned} {{\,\mathrm{\mathbb {P}}\,}}\left[ \exists A \in {\mathcal S}: K_{A} \subseteq {\varvec{G}}_{n,p} \cup K_{B} \text { and } A \subseteq {\varvec{U}}_{n,q} \cup B \right] > 1-\varepsilon . \end{aligned}$$

The set *B* is called *core*.

Clearly, a $(p,1,\varepsilon )$-clique sunflower is a $(p,\varepsilon )$-clique sunflower. By taking $q=1$ in the following lemma, and observing that $s_\ell (\log (1/\varepsilon )) \leqslant \log (1/\varepsilon )+1/2 \leqslant 2\log (1/\varepsilon )$ for $\varepsilon \leqslant e^{-1/2}$, we obtain Lemma 11.

#### Lemma 23

For all $\ell \in \{1,\dots ,n\}$ and $$S \subseteq \left( {\begin{array}{c}[n]\\ \ell \end{array}}\right) $$, if $$\left|{\mathcal S}\right| > s_\ell (\log (1/\varepsilon )) \cdot (1/q)^\ell (1/p)^{\left( {\begin{array}{c}\ell \\ 2\end{array}}\right) }$$, then ${\mathcal S}$ contains a $(p,q,\varepsilon )$-clique sunflower.

#### Proof

By induction on $\ell$. In the base case $\ell =1$, we have by independence that

$$\begin{aligned} {{\,\mathrm{\mathbb {P}}\,}}[ \forall A \in {\mathcal S}: K_A \nsubseteq {\varvec{G}}_{n,p} \text { or } A \nsubseteq {\varvec{U}}_{n,q} ]&= {{\,\mathrm{\mathbb {P}}\,}}[ \forall A \in {\mathcal S}: A \nsubseteq {\varvec{U}}_{n,q} ]\\&= \prod _{A \in S} {{\,\mathrm{\mathbb {P}}\,}}[ A \nsubseteq {\varvec{U}}_{n,q} ]\\&= (1-q)^{|S|} < (1-q)^{\ln (1/\varepsilon )/q} \leqslant e^{-\ln (1/\varepsilon )} = \varepsilon . \end{aligned}$$

Thus ${\mathcal S}$ is itself a $(p,q,\varepsilon )$-clique sunflower.

Let now $\ell \geqslant 2$ and assume that the claim holds for $t \in \left\{ 1,\dots ,\ell -1\right\}$. For convenience, let

$$\begin{aligned} c_j := s_j(\log (1/\varepsilon )), \end{aligned}$$

for every $$j \in \left\{ 0,1,\dots ,\ell -1\right\} $$.

*Case 1.* There exists $j \in \{1,\dots ,\ell -1\}$ and $$B \in \left( {\begin{array}{c}[n]\\ j\end{array}}\right) $$ such that

$$\begin{aligned} |\{A \in {\mathcal S}: B \subseteq A\}| \geqslant c_{\ell -j} (1/qp^j)^{\ell -j}(1/p)^{\left( {\begin{array}{c}\ell -j\\ 2\end{array}}\right) }. \end{aligned}$$

Let $${\mathcal T}= \{A \setminus B : A \in {\mathcal S}\text { such that } B \subseteq A\} \subseteq \left( {\begin{array}{c}[n]\\ \ell -j\end{array}}\right) $$. By the induction hypothesis, there exists a $(p,qp^j,\varepsilon )$-clique sunflower ${\mathcal T}' \subseteq {\mathcal T}$ with core a *D* satisfying $$D \in \left( {\begin{array}{c}[n] \setminus B\\ <\ell -j\end{array}}\right) $$. We will now show that ${\mathcal S}' := \left\{ B \cup C : C \in {\mathcal T}'\right\} \subseteq {\mathcal S}$ is a $(p,q,\varepsilon )$-clique sunflower contained in ${\mathcal S}$ with core ${B \cup D}$. We have

$$\begin{aligned} {{\,\mathrm{\mathbb {P}}\,}}[&\forall {A} \in {\mathcal S}': K_{A} \nsubseteq&{\varvec{G}}_{n,p} \cup K_{B \cup D} \text { or } A \nsubseteq {\varvec{U}}_{n,q} \cup B \cup D ]\\&= {{\,\mathrm{\mathbb {P}}\,}}[ \forall C \in {\mathcal T}':&K_{B \cup C} \nsubseteq {\varvec{G}}_{n,p} \cup K_{B \cup D} \text { or } B \cup C \nsubseteq {\varvec{U}}_{n,q} \cup B \cup D ]\\&= {{\,\mathrm{\mathbb {P}}\,}}[ \forall C \in {\mathcal T}':&K_{B \cup C} \nsubseteq {\varvec{G}}_{n,p} \cup K_{B \cup D} \text { or } C \nsubseteq {\varvec{U}}_{n,q} \cup D ]\\&= {{\,\mathrm{\mathbb {P}}\,}}[ \forall C \in {\mathcal T}':&K_C \nsubseteq {\varvec{G}}_{n,p} \cup K_D \text { or } \\&\&C \nsubseteq \big \{v \in {\varvec{U}}_{n,q} : \{v,w\} \in E({\varvec{G}}_{n,p}) \text { for all } w \in B\big \} \cup D ]\\&\leqslant {{\,\mathrm{\mathbb {P}}\,}}[ \forall C \in {\mathcal T}':&K_C \nsubseteq {\varvec{G}}_{n,p} \cup K_D \text { or } C \nsubseteq {\varvec{U}}_{n,qp^j} \cup D ]\\&< \varepsilon .&\end{aligned}$$

Therefore, ${\mathcal S}'$ is a $(p,q,\varepsilon )$-clique sunflower contained in ${\mathcal S}$.

*Case 2.* For all $j \in \{1,\dots ,\ell -1\}$ and $$B \in \left( {\begin{array}{c}[n]\\ j\end{array}}\right) $$, we have

$$\begin{aligned} |\{A \in {\mathcal S}: B \subseteq A\}| \leqslant c_{\ell -j}(1/qp^j)^{\ell -j}(1/p)^{\left( {\begin{array}{c}\ell -j\\ 2\end{array}}\right) }. \end{aligned}$$

In this case, we show that the bound of the lemma holds with $B = \emptyset$. Let

$$\begin{aligned} \mu&:=\left|{\mathcal S}\right| q^\ell p^{\left( {\begin{array}{c}\ell \\ 2\end{array}}\right) } > c_\ell , \\ \overline{\varDelta }&:=\sum _{j=1}^{\ell -1} \sum _{(A,A') \in {\mathcal S}^2 \,:\, |A \cap A'| = j} q^{2\ell -j} p^{2\left( {\begin{array}{c}\ell \\ 2\end{array}}\right) - \left( {\begin{array}{c}j\\ 2\end{array}}\right) }. \end{aligned}$$

Note that $\overline{\varDelta }$ excludes $j = \ell$ from the sum, which corresponds to pairs $(A,A')$ such that $A = A'$, in which case the summand becomes $\mu$. In other words, the number $\varDelta$ of Janson’s inequality (Lemma 21) satisfies $\varDelta = \mu + \overline{\varDelta }$. Janson’s Inequality now gives the following bound:

4 $$\begin{aligned} {{\,\mathrm{\mathbb {P}}\,}}[\forall A \in {\mathcal S}: K_A \nsubseteq {\varvec{G}}_{n,p} \text { or } A \nsubseteq {\varvec{U}}_{n,q} ] \leqslant \exp \left( - \frac{\mu ^2}{\mu +\overline{\varDelta }} \right) . \end{aligned}$$

We bound $\overline{\varDelta }$ as follows:

$$\begin{aligned} \overline{\varDelta }&\leqslant \sum _{j=1}^{\ell -1} q^{2\ell -j} p^{2\left( {\begin{array}{c}\ell \\ 2\end{array}}\right) - \left( {\begin{array}{c}j\\ 2\end{array}}\right) } \sum _{B \in \left( {\begin{array}{c}[n]\\ j\end{array}}\right) } |\{A \in S : B \subseteq A\}|^2 \\&\leqslant \sum _{j=1}^{\ell -1} q^{2\ell -j} p^{2\left( {\begin{array}{c}\ell \\ 2\end{array}}\right) - \left( {\begin{array}{c}j\\ 2\end{array}}\right) } \sum _{B \in \left( {\begin{array}{c}[n]\\ j\end{array}}\right) } |\{A \in S : B \subseteq A\}| \cdot c_{\ell -j}(1/q)^{\ell -j}(1/p)^{\left( {\begin{array}{c}\ell -j\\ 2\end{array}}\right) }\\&\leqslant q^{\ell }p^{\left( {\begin{array}{c}\ell \\ 2\end{array}}\right) } \sum _{j=1}^{\ell -1} c_{\ell -j} \sum _{B \in \left( {\begin{array}{c}[n]\\ j\end{array}}\right) } |\{A \in S : B \subseteq A\}|\\&= q^{\ell }p^{\left( {\begin{array}{c}\ell \\ 2\end{array}}\right) } \sum _{j=1}^{\ell -1} c_{\ell -j} \sum _{A \in S} \sum _{B \in \left( {\begin{array}{c}A\\ j\end{array}}\right) } 1\\&= |S| q^{\ell }p^{\left( {\begin{array}{c}\ell \\ 2\end{array}}\right) } \sum _{j=1}^{\ell -1} \left( {\begin{array}{c}\ell \\ j\end{array}}\right) c_{\ell -j} \\&= \mu \sum _{j=1}^{\ell -1} \left( {\begin{array}{c}\ell \\ j\end{array}}\right) c_j = \mu \sum _{j=0}^{\ell -1} \left( {\begin{array}{c}\ell \\ j\end{array}}\right) c_j - \mu . \end{aligned}$$

Therefore,

$$\begin{aligned} \frac{\mu ^2}{\mu + \overline{\varDelta }} \geqslant \frac{\mu }{\sum _{j=0}^{\ell -1} \left( {\begin{array}{c}\ell \\ j\end{array}}\right) c_j } = \frac{\mu }{c_\ell /(\log (1/\varepsilon ))} > \log (1/\varepsilon ). \end{aligned}$$

Finally, from (4) we get

$$\begin{aligned} {{\,\mathrm{\mathbb {P}}\,}}[\forall A \in {\mathcal S}: K_A \nsubseteq {\varvec{G}}_{n,p} \text { or } A \nsubseteq {\varvec{U}}_{n,q} ] \leqslant \exp \left( - \frac{\mu ^2}{\mu +\overline{\varDelta }} \right) < \varepsilon . \end{aligned}$$

Therefore, the family ${\mathcal S}$ is a $(p,q,\varepsilon )$-clique sunflower with an empty core. $\square$

## Monotone Arithmetic Circuits

In this section, we give a short and simple proof of a truly exponential ($\exp (\varOmega (n))$) lower bound for real monotone arithmetic circuits computing a multilinear *n* variate polynomial. Real monotone arithmetic circuits are arithmetic circuits over the reals that use only positive numbers as coefficients. As we shall see, the lower bound argument holds for a general family of multilinear polynomials constructed in a very natural way from error correcting codes, and the similarities to the hard function used by Harnik and Raz in the Boolean setting is quite evident (see Sect. 2.2). In particular, our lower bound just depends on the rate and relative distance of the underlying code. We note that exponential lower bounds for monotone arithmetic circuits are not new, and have been known since the 80’s with various quantitative bounds. More precisely, Jerrum and Snir proved an $\exp (\varOmega (\sqrt{n}))$ lower bound for an *n* variate polynomial in [15]. This bound was subsequently improved to a lower bound of $\exp (\varOmega (n))$ by Raz and Yehudayoff in [22], via an extremely clever argument, which relied on deep and beautiful results on character sums over finite fields. A similar lower bound of $\exp (\varOmega (n))$ was shown by Srinivasan [26] using more elementary techniques building on a work of Yehudayoff [30]. In a recent personal communication Igor Sergeev pointed out to us that truly exponential lower bounds for monotone arithmetic circuits had also been proved in the 1980’s in the erstwhile Soviet Union by several authors, including the works of Kasim-Zade, Kuznetsov and Gashkov. We refer the reader to [10] for a detailed discussion on this line of work.

We show a similar lower bound of $\exp (\varOmega (n))$ via a simple and short argument, which holds in a somewhat general setting. Our contribution is just the simplicity, the (lack of) length of the argument and the observation that it holds for families of polynomials that can be constructed from any sufficiently *good* error correcting codes.

### Definition 11

*(Monotone, multilinear, homogeneous)* A real polynomial is said to *monotone* if all of its coefficients are positive. A real arithmetic circuit is said to be *monotone* if it uses only positive numbers as coefficients. A polynomial *P* is said to be *multilinear* if the degree of each variable of *P* is at most 1 in all of the monomials of *P*. A polynomial *P* is said to be *homogeneous* if all the monomials of *P* have the same degree. An arithmetic circuit *C* is said to be to *homogeneous* (*multilinear*) if the polynomial computed in each of the gates of *C* is homogeneous (multilinear).

### Definition 12

*(From sets of vectors to polynomials)* Let ${C} \subseteq \mathbb {F}_q^n$ be an arbitrary subset of $\mathbb {F}_q^n$. Then, the polynomial $P_{C}$ is a multilinear homogeneous polynomialof degree *n* on *qn* variables $\{x_{i, j} : i \in [q], j \in [n]\}$ and is defined as follows:

$$\begin{aligned} P_C = \sum _{c \in C} \prod _{j \in [n]} x_{c(j), j} \, . \end{aligned}$$

Here, *c*(*j*) is the $j^{th}$ coordinate of *c* which is an element of $\mathbb {F}_q$, which we bijectively identify with the set [*q*].

Here, we will be interested in the polynomial $P_C$ when the set *C* is a *good* code, i.e it has high rate and high relative distance. The following observation summarizes the properties of $P_C$ and relations between the properties of *C* and $P_C$.

### Observation 6

(Codes vs Polynomials) Let *C* be any subset of $\mathbb {F}_q^n$ and let $P_C$ be the polynomial as defined in Definition 12. Then, the following statements are true:

- $P_C$ is a multilinear homogeneous polynomial of degree equal to *n* with every coefficient being either 0 or 1.
- The number of monomials with non-zero coefficients in $P_C$ is equal to the cardinality of *C*.
- If any two distinct vectors in *C* agree on at most *k* coordinates (i.e. *C* is a code of distance $n-k$), then the intersection of the support of any two monomials with non-zero coefficients in $P_C$ has size at most *k*.

The observation immediately follows from Definition 12. We note that we will work with monotone arithmetic circuits here, and hence will interpret the polynomial $P_C$ as a polynomial over the field of real numbers.

We now prove the following theorem, which essentially shows that for every code *C* with sufficiently good distance, any monotone arithmetic circuit computing $P_C$ must essentially compute it by computing each of its monomials separately, and taking their sum.

### Theorem 7

If any two distinct vectors in *C* agree on at most $n/3-1$ locations, then any monotone arithmetic circuit for $P_C$ has size at least |*C*|.

The proof of this theorem crucially uses the following well known structural lemma about arithmetic circuits. This lemma also plays a crucial role in the other proofs of exponential lower bounds for monotone arithmetic circuits (e.g. [15, 22, 26, 30]).

### Lemma 24

(See Lemma 3.3 in [22]) Let *Q* be a homogeneous multilinear polynomial of degree *d* computable by a homogeneous arithmetic circuit of size *s*. Then, there are homogeneous polynomials $g_0, g_1, g_2, \ldots , g_s, h_0, h_1, h_2, \ldots , h_s$ of degree at least *d*/3 and at most $2d/3-1$ such that

$$\begin{aligned} Q = \sum _{i = 0}^{s} g_i\cdot h_i \, . \end{aligned}$$

Moreover, if the circuit for *Q* is monotone, then each $g_i$ and $h_i$ is multilinear, variable disjoint and each one their non-zero coefficients is a positive real number.

We now use this lemma to prove Theorem 7.

### Proof of Theorem 7

Let *B* be a monotone arithmetic circuit for $P_C$ of size *s*. We know from Observation 6 that $P_C$ is a multilinear homogeneous polynomial of degree equal to *n*. This along with the monotonicity of *B* implies that *B* must be homogeneous and multilinear since there can be no cancellations in *B*. Thus, from (the moreover part of) Lemma 24 we know that $P_C$ has a monotone decomposition of the form

$$\begin{aligned} P_C = \sum _{i = 0}^s g_i\cdot h_i \, , \end{aligned}$$

where, each $g_i$ and $h_i$ is multilinear, homogeneous with degree between *n*/3 and $2n/3-1$, $g_i$ and $h_i$ are variable disjoint. We now make the following claim.

### Claim

Each $g_i$ and $h_i$ has at most one non-zero monomial.

We first observe that the claim immediately implies theorem 7: since every $g_i$ and $h_i$ has at most one non-zero monomial, their product $g_ih_i$ is just a monomial. Thus, the number of summands *s* needed in the decomposition above must be equal to the number of monomials in $P_C$, which is equal to |*C*| from the second item in Observation 6. $\square$

We now prove the Claim.

### Proof of Claim

The proof of the claim will be via contradiction. To this end, let us assume that there is an $i \in \{0, 1, 2, \ldots , s\}$ such that $g_i$ has at least two distinct monomials with non-zero coefficients and let $\alpha$ and $\beta$ be two of these monomials. Let $\gamma$ be a monomial with non-zero coefficient in $h_i$ . Since $h_i$ is homogeneous with degree between *n*/3 and $2n/3-1$, we know that the degree of $\gamma$ is at least *n*/3. Since we are in the monotone setting, we also know that each non-zero coefficient in any of the $g_j$ and $h_j$ is a positive real number. Thus, the monomials $\alpha \cdot \gamma$ and $\beta \cdot \gamma$ which have non-zero coefficients in the product $g_i\cdot h_i$ must have non-zero coefficient in $P_C$ as well (since a monomial once computed cannot be cancelled out). But, the supports of $\alpha \gamma$ and $\beta \gamma$ overlap on $\gamma$ which has degree at least *n*/3. This contradicts the fact that no two distinct monomials with non-zero coefficients in $P_C$ share a sub-monomial of degree at least *n*/3 from the distance of *C* and the third item in Observation 6. $\square$

Theorem 7 when instantiated with an appropriate choice of the code *C*, immediately implies an exponential lower bound on the size of monotone arithmetic circuits computing the polynomial $P_C$. Observe that the total number of variables in $P_C$ is $N = qn$ and therefore, for the lower bound for $P_C$ to be of the form $\exp (\varOmega (N))$, we would require *q*, the underlying field size to be a constant. In other words, for any code of relative distance at least 2/3 over a constant size alphabet which has exponentially many code words, we have a truly exponential lower bound.

The following theorem of Garcia and Stichtenoth [9] implies an explicit construction of such codes. The statement below is a restatement of their result by Cohen et al. [7].

### Theorem 8

([9] and [27]) Let *p* be a prime number and let $m\in \mathbb {N}$ be even. Then, for every $0<\rho < 1$ and a large enough integer *n*, there exists an explicit rate $\rho$ linear error correcting block code $C: \mathbb {F}_{p^m}^n \rightarrow \mathbb {F}_{p^m}^{n/\rho }$ with distance

$$\begin{aligned} \delta \geqslant 1- \rho - \frac{1}{p^{m/2} - 1} \, . \end{aligned}$$

The theorem has the following immediate corollary.

### Corollary 2

For every large enough constant *q* which is an even power of a prime, and for all large enough *n*, there exist explicit construction of codes $C \subseteq \mathbb {F}_q^n$ which have relative distance at least 2/3 and $|C| \geqslant \exp (\varOmega (n))$.

By an explicit construction here, we mean that given a vector *v* of length *n* over $\mathbb {F}_q$, we can decide in deterministic polynomial time if $v \in C$. In the arithmetic complexity literature, a polynomial *P* is said to be explicit, if given the exponent vector of a monomial, its coefficient in *P* can be computed in deterministic polynomial time. Thus, if a code *C* is explicit, then the corresponding polynomial $P_C$ is also explicit in the sense described above. Therefore, we have the following corollary of Corollary 2 and Theorem 7.

### Corollary 3

There exists an explicit family $\{P_n\}$ of homogeneous multilinear polynomials such that for every large enough *n*, any monotone arithmetic circuit computing the *n* variate polynomial $P_n$ has size at least $\exp (\varOmega (n))$.

## Further Directions

In this paper, we obtained the first monotone circuit lower bound of the form $\exp (\varOmega (n^{1/2}/\log n))$ for an explicit *n*-bit monotone Boolean function. It’s natural to ask if we can do better. Ideally, we would like to achieve a truly exponential bound for Boolean monotone circuits, like the one achieved for arithmetic monotone circuits in Sect. 4. However, as discussed in Sect. 2.8, the $\sqrt{n}$ exponent seems to be at the limit of what current techniques can achieve.

An important open-ended direction is to develop sharper techniques for proving monotone circuit lower bounds. Sticking to the approximation method, it is not yet known whether there exists another “sunflower-type” notion which still allows for good approximation bounds and yet admits significantly better bounds than what is possible for robust sunflowers.

One approach can be to try to weaken the requirement of the core, and ask only that the core of a “sunflower-type” set system ${\mathcal {F}}$ is properly contained in one of the elements of ${\mathcal {F}}$. A weaker notion of robust sunflowers with this weakened core could still be used succesfully in the proof of the lower bound of Sect. 2, but it’s not yet clear whether this weaker notion admits stronger bounds or not.

Moreover, perhaps developing specialised sunflowers for specific functions, such as done for $\mathsf {Clique}_{k,n}$ in Sect. 3, could help here. One could also consider distributions which are not *p*-biased, as perhaps better bounds are possible in different regimes.

Finally, as noted before, our proof of the clique-sunflower lemma follows the approach of Rossman in [24]. We expect that a proof along the lines of the work of Alweiss, Lovett, Wu and Zhang [3] and Rao [21] should give us an even better bound on the size of set systems without clique-sunflowers, removing the $\ell !$ factor. This would extend our $n^{\varOmega (\delta ^2 k)}$ lower bound to $k \leqslant n^{1/2-\delta }$.

## Proof of Theorem 3

We say that a family ${\mathcal {F}}$ of sets is *r*-*spread* if there are most $\left|{\mathcal {F}}\right|/r^{\left|T\right|}$ sets in ${\mathcal {F}}$ containing any given non-empty set *T*. The following theorem is a *p*-biased variant of the main technical lemma of Rao [21]. A full proof is given in the appendix of [6].

### Theorem 9

(Theorem 3 of [6]) There exists a constant $B > 0$ such that the following holds for all $p, \varepsilon \in (0,1/2]$ and all positive integers $\ell$. Let $r = B \log (\ell /\varepsilon )/p$. Let ${\mathcal {F}}$ be a *r*-spread $\ell$-uniform family of subsets of [*n*] such that $\left|{\mathcal {F}}\right| \geqslant r^{\ell }$. Then ${{\,\mathrm{\mathbb {P}}\,}}_{{\varvec{W}}\subseteq _p [n]} [\exists F \in {\mathcal {F}} : F \subseteq {\varvec{W}}] > 1-\varepsilon$.

We now combine Theorem 9 with the main argument of the proof of Theorem 4.4 of [24] to finish the proof of Theorem 3.

### Proof of Theorem 3

The proof is by induction on $\ell$. When $\ell =1$, we have

$$\begin{aligned} {{\,\mathrm{\mathbb {P}}\,}}[\forall F \in {\mathcal {F}} : F \not \subseteq {\varvec{W}}] = (1-p)^{\left|{\mathcal {F}}\right|} \leqslant e^{-p \left|{\mathcal {F}}\right|} < \varepsilon . \end{aligned}$$

Therefore, ${\mathcal {F}}$ itself is a $(p,\varepsilon )$-robust sunflower. We now suppose $\ell > 1$ and that the result holds for every $t \in [\ell -1]$. For a set $T \subseteq [n]$, let ${\mathcal {F}}_T = \left\{ F \setminus T : F \in {\mathcal {F}}, T \subseteq F\right\}$. Let $r = B \log (\ell /\varepsilon )/p$, where *B* is the constant of Theorem 9.

*Case 1.* The family ${\mathcal {F}}$ is not *r*-spread. By definition, there exists a nonempty set $T \subseteq [n]$ such that $\left|{\mathcal {F}}_T\right| > \left|{\mathcal {F}}\right|/r^{\left|T\right|} \geqslant r^{\ell -\left|T\right|}$. By induction, the family ${\mathcal {F}}_T$ contains a $(p,\varepsilon )$-robust sunflower ${\mathcal {F}}'$. It is easy to see that $\left\{ F \cup T : F \in {\mathcal {F}}'\right\}$ is a $(p,\varepsilon )$-robust sunflower contained in ${\mathcal {F}}$.

*Case 2.* The family ${\mathcal {F}}$ is *r*-spread. Therefore, from Theorem 9, it follows that ${\mathcal {F}}$ is itself a $(p,\varepsilon )$-robust sunflower. $\square$

## Footnotes

1 Stasys Jukna (personal communication) observed that Andreev’s bound [4] can be improved to $2^{\varOmega ((n/\sqrt{\log n})^{1/3})}$ using the lower bound criterion of [16].

2 Robust sunflowers were called *quasi-sunflowers* in [11, 17, 18, 24] and *approximate sunflowers* in [19]. Following Alweiss *et al* [3], we adopt the new name *robust sunflower*.

3 Rao’s bound is also slightly stronger in the following sense. He shows that, if the random set ${\varvec{W}}$ is chosen uniformly at random among all sets of size $\lfloor np\rfloor$, then we also have ${{\,\mathrm{\mathbb {P}}\,}}\left[ \exists F \in {\mathcal {F}}: F \subseteq {\varvec{W}}\cup Y \right] > 1-\varepsilon$. However, for our purposes, the *p*-biased case will suffice.

4 Here, “random minterm” means an input from the distribution ${\varvec{Y}}$, which correlates highly with the minterms of $f_\text {HR}$.

5 Formally, our approximators have at most $O(c \log n)^\ell$ terms of width $\ell$ (Lemma 5), and no terms of width larger than *c*/2 (by trimming).
